# Materials Dual-Source Knowledge Retrieval-Augmented Generation for Local Large Language Models in Photocatalysts

**DOI:** 10.1021/acs.jcim.5c01941

**Published:** 2025-12-01

**Authors:** Wataru Takahara, Yuichi Yamaguchi, Mai Ogano, Fuga Kakami, Yosuke Harashima, Tomoaki Takayama, Shogo Takasuka, Akihiko Kudo, Mikiya Fujii

**Affiliations:** † Division of Materials Science, 12708 Nara Institute of Science and Technology, Nara-ken, Ikoma-shi 630-0192, Japan; ‡ Department of Applied Chemistry, Faculty of Science, 26413 Tokyo University of Science, Shinjuku-ku, Tokyo 162-8601, Japan; § Carbon Value Research Center, Research Institute for Science & Technology, Tokyo University of Science, Noda-shi, Chiba-ken 278-8510, Japan; ∥ Data Science Center, Nara Institute of Science and Technology, Nara-ken, Ikoma-shi 630-0192, Japan; ⊥ Center for Material Research Platform, Nara Institute of Science and Technology, Nara-ken, Ikoma-shi 630-0192, Japan

## Abstract

Large language models (LLMs) have the potential to serve as collaborative assistants in scientific research. However, adapting them to specialized domains is difficult because it requires the integration of domain-specific knowledge. We propose Materials Dual-Source Knowledge Retrieval-Augmented Generation (MDSK-RAG), a retrieval-augmented generation (RAG) framework that enables domain specialization of LLMs for materials development under fully offline (no-Internet) operation to ensure data confidentiality. The framework unifies two complementary knowledge sources, experimental CSV data (practical knowledge) and scientific PDF literature (theoretical insights), by converting tabular records into template-based text, retrieving relevant passages from each source, summarizing them with a local LLM, and merging the summaries with the user query prior to generation. As a case study, we applied the framework to metal-sulfide photocatalysts using 740 in-house experimental records and 20 scientific PDFs. We evaluated the framework on a benchmark consisting of 14 expert-defined questions and used two-sided Wilcoxon signed-rank tests for paired comparisons. Models with fewer than 10 billion parameters were executed on a laptop, whereas larger models were run on a dedicated local server; the cloud-based LLM (GPT-4o) was evaluated via the cloud service. For practical deployment, gemma-2-9b-it (<10 billion parameters) was chosen as the primary local model; we additionally tested Qwen2.5-7B-Instruct and a larger gemma-2-27b-it to assess model choice and scalability. For gemma-2-9b-it, the framework increased the median cosine similarity to expert reference answers from 0.63 to 0.71, an absolute increase of 0.08 (corresponding to a relative percentage gain of 12.70%; Wilcoxon signed-rank test statistic: *W* = 14.0, two-sided *p*-value: *p* = 1.34 × 10^–2^) and improved the median expert 5-point rating from 2 to 3, an absolute increase of 1 point (corresponding to a relative percentage gain of 50.00%; Wilcoxon signed-rank test statistic: *W* = 3.5, two-sided *p*-value: *p* = 7.00 × 10^–3^). For reasoning-type questions, incomplete context retrieved by MDSK-RAG sometimes disrupted the model’s reasoning process and led to incorrect conclusions, indicating remaining room for improvement. Comparable, statistically significant improvements were observed for the other local models (Qwen2.5-7B-Instruct and a larger gemma-2-27b-it) between conditions with and without the framework in the evaluation by cosine similarity to expert reference answers. In comparison to a cloud-based LLM, the gemma-2-9b-it with the framework outperformed GPT-4o. In this case study, the framework effectively incorporated practical experimental knowledge and theoretical literature into local LLM responses, improving accuracy for domain-specific queries. The framework presented here offers a practical and extensible adaptation of local LLMs to domain-specific scientific research.

## Graphical abstract

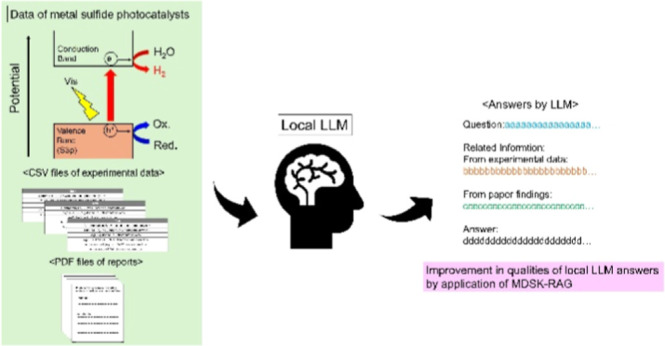

## 1 Introduction

Recently, large language models (LLMs) have been rapidly adopted in various scientific fields and applied to a wide range of tasks, such as code generation,^1^ data analysis,^2^ and literature summarization.^3^ Recent advances in the adoption of LLMs in material studies have focused on two primary applications. The first is an interface between humans and autonomous experimental systems.^4^ The second is a “co-scientist,” that is, an artificial intelligence (AI) system that collaborates with human scientists in the course of scientific discovery.^5,6^ Two types of LLMs exist: cloud-based LLMs provided as services on the internet, such as ChatGPT,^7^ developed by OpenAI Inc., and local LLMs that run on user-controlled infrastructure without a connection to the internet. Although the former exhibits high performance, its use with confidential data is restricted because confidential data must not be uploaded to the cloud. Despite the limited resources of local LLMs, they offer key advantages in that data confidentiality is assured. This advantage makes local LLMs particularly attractive for scientific domains that handle proprietary and unpublished data. Motivated by this advantage, we adopted a local LLM in the present study to explore its applicability in materials development. Previous studies explored the use of local LLMs as assistants in materials development. Bai et al. demonstrated that local LLMs could assist in tasks related to metal-organic frameworks, such as the extraction of information, generation of synthesis conditions, and generation of code, using domain-relevant prompts.^5^ Choi et al. fine-tuned (specifically, low-rank adaptation^8^) a base model with 1883 in-house synthesis records for application in quantum dot material studies. Their study showed that domain-adapted LLMs can support synthesis planning and facilitate materials discovery through multiobjective optimization.^6^ These studies illustrate the potential of local LLMs and highlight practical challenges, particularly the need for time-consuming prompt engineering or additional fine-tuning.

Domain specialization is crucial for the effective application of LLMs in scientific fields. However, incorporating domain-specific knowledge into LLMs is difficult. Several approaches have been proposed to achieve domain adaptation, including additional training (e.g., continued pretraining, fine-tuning), prompt engineering (e.g., few-shot learning^9^), and retrieval-augmented generation (RAG).^10^ Additional training involves retraining LLMs with domain-specific data to improve their capabilities in specific scientific fields. This approach requires significant computational resources and large amounts of high-quality data. However, as Butler et al. note, ″in chemistry or materials science we are often limited to hundreds or thousands, if not fewer, high-quality data points.″^11^ This limitation arises directly from the real-world cost and effort of generating experimental data: each reliable data point commonly requires sample synthesis or fabrication, purification, and access to specialized instrumentation, often operated by skilled personnel. Unlike domains where large-scale passive data collection is available, materials science faces structural barriers to assembling very large and labeled data. Thus, it remains challenging to integrate a limited amount of new knowledge that emphasizes the detailed aspects of a specific domain.

Prompt engineering, also known as “in-context learning” is a technique in which domain-specific context, such as instructions and examples, is directly provided to LLMs as part of the input prompts to guide their responses. Few-shot learning is a method in which multiple examples are provided to LLMs as input prompts. Both prompt engineering and few-shot learning have limitations in dynamically incorporating domain-specific knowledge because of the fixed and example-based guidance within the prompt. By contrast, the RAG framework enables LLMs to access and retrieve domain-specific knowledge from an external knowledge base in real time, which allows them to integrate newly updated information without modifying the model itself. This is particularly important in materials science, where each material system often involves specific terms, notations, and domain conventions. RAG-based frameworks can adapt more effectively to diverse scientific subfields by directly retrieving domain-specific expressions from curated data. RAG adoption is increasing, as shown by recent reports demonstrating modular and reproducible architecture based on RAG,^12^ graph-based RAG framework,^13^ and multimodal RAG framework.^14^ Concurrently, RAG applications in materials science are beginning to appear examples such as RAG frameworks that build structured databases from published papers,^15^ agent-driven RAG systems that convert curated literature into experiment-ready recommendations,^16^ and RAG approaches that reduce hallucinations.^17^ Here, many of these materials-domain studies primarily rely on openly available sources, and often lack the detailed experimental metadata including records of negative results that are typically kept only in-house. In our view, an effective domain adaptation when applying RAG to materials development requires two information sources. The first is publicly recognized scientific knowledge. This knowledge is essential for understanding the target material system. The second is detailed records of the experimental conditions. These records should also contain information about failed trials, which are rarely disclosed in publications. The former refers to the peer-reviewed scientific literature typically available to the public. The latter refers to the in-house experimental data that are typically not shared externally. Literature-based data are presented in textual form; thus, LLMs can process them more easily. In contrast, the experimental data are formatted as tables. LLMs are generally known to struggle with reasoning tasks involving structured tabular data.^18^

In this study, we present a framework called materials dual-source knowledge RAG (MDSK-RAG; pronounced “Em-desk-RAG”). This framework enables domain adaptation of a local LLM by integrating two distinct knowledge sources: experimental CSV data (practical knowledge) and scientific PDF literature (theoretical insights). The name “MDSK” is inspired by the idea of a “desk” that serves as a unified workspace for handling both sources in an integrated manner. The framework allows the knowledge base to be flexibly updated, such that newly published or internally generated information can be incorporated without fine-tuning the model itself.

In the present study, the framework was applied to construct a local LLM that specializes in metal-sulfide photocatalysts as a case study. Photocatalysts have gained increasing interest for their relevance to carbon neutrality and the Sustainable Development Goals (SDGs). These metal-sulfide materials have garnered considerable attention owing to their potential applications in water splitting and CO_2_ reduction.^19–22^ One important reason for this is that several of these materials respond to visible light and produce hydrogen with relatively high efficiency. However, discovering novel and efficient photocatalysts remains a major challenge, as it requires not only a deep theoretical understanding but also substantial experimental knowledge, such as synthesis conditions and structural stability. In this domain, five attributes are especially important—composition, crystal-structure type, synthesis method, reaction conditions, and hydrogen-evolution activity—since these variables are directly linked to the physicochemical mechanisms that determine photocatalytic performance. Below we summarize the background reasoning.^23^ As shown in Figure 1, the band structure of metal sulfides consists of valence and conduction bands filled with electrons and no electrons, respectively. The differential energy between those bands is called a band gap. Electrons in a valence band are excited to a conduction band when a metal-sulfide photocatalyst absorbs light whose energy is larger than the bandgap. The band levels and the band gap are mainly determined by the constituent elements (compositions of photocatalysts) and the crystal structure. As the next step, migration of the photogenerated electrons and holes to the surface through a cocatalyst functioning as an active site is necessary to react with water. The synthesis method and the reaction conditions greatly affect the crystallinity, particle size, surface area, and defect formation. These factors affect the probability of which photogenerated carriers migrate to the surface of the photocatalyst.

**Figure 1:**
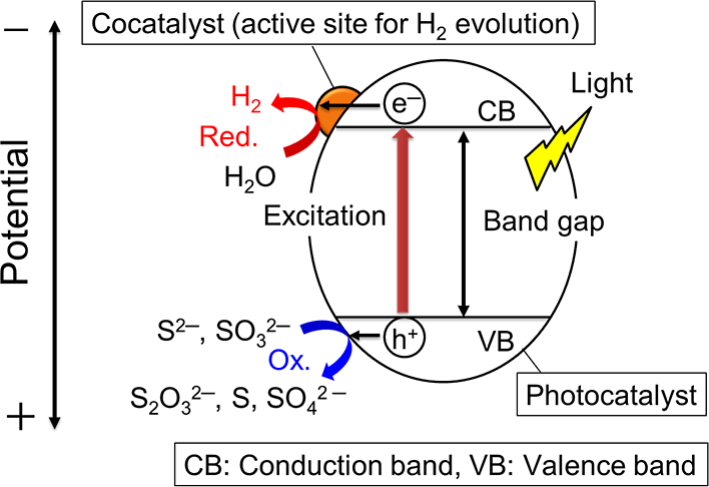
Photocatalytic hydrogen evolution over metal-sulfide photocatalysts from an aqueous solution containing S^2–^ and SO_3_^2–^ ions as sacrificial reagents.

Based on this background, the present study aims to enable a local LLM to generate informative responses regarding candidate materials and synthesis strategies, without relying on fine-tuning or cloud-based processing.

## 2 Methods

### 2.1 Data Preparation

All experimental data used in the present study were curated from experiments conducted by Kudo et al., who are coauthors, for developing various metal-sulfide photocatalysts. We prepared a tabular CSV file containing 740 experimental records of metal-sulfide photocatalysts, along with 13 relevant published articles^19,24–35^ and three review papers^36–38^ from the Kudo group in PDF format. We also included a published article on metal-sulfide photocatalysts^39^ and three publications (a published article^40^ and two review papers^41,42^ ) reviewing various photocatalytic material groups including metal-sulfide photocatalysts. With these additions, the total number of PDF knowledge documents used in the present study was 20. Photocatalytic performance is strongly influenced by experimental parameters, and direct cross-study quantitative comparison is often problematic and can introduce noise into information retrieval. Therefore, published articles outside the Kudo group were selected with particular care by domain experts to ensure relevance to the present data set. The tabular data in the CSV file comprise columns representing the photocatalyst materials, experimental conditions, and photocatalytic activity. Each record comprises the following columns: one column for the photocatalyst type, which refers to the host materials such as ZnS, La_2_S_3_, and CuInS_2_; 100 columns for experimental conditions including both direct numerical values and one-hot encoded binary indicators, which denote the presence or absence of specific experimental conditions; 15 columns representing crystal structure types as one-hot vectors; and one column representing hydrogen evolution reaction rates in units of μmol h^–1^.

To make the tabular experimental data in the CSV file more interpretable by LLMs and to treat the data in the CSV and PDF files equivalently, each record in the tabular experimental data was converted into text following the flowchart outlined in Figure 2. The sentence templates used for conversion at each step in the flowchart are described in Box 1. The experimental data in the present study are of a realistic size for materials-science studies, as noted by Butler et al. We recognize that template-based conversion of tabular data may introduce representational bias and face scalability limits. To mitigate these risks in the present study, the templates and flowchart were designed by domain experts to comprehensively cover the experimental patterns of metal-sulfide photocatalysts.

**Figure 2:**
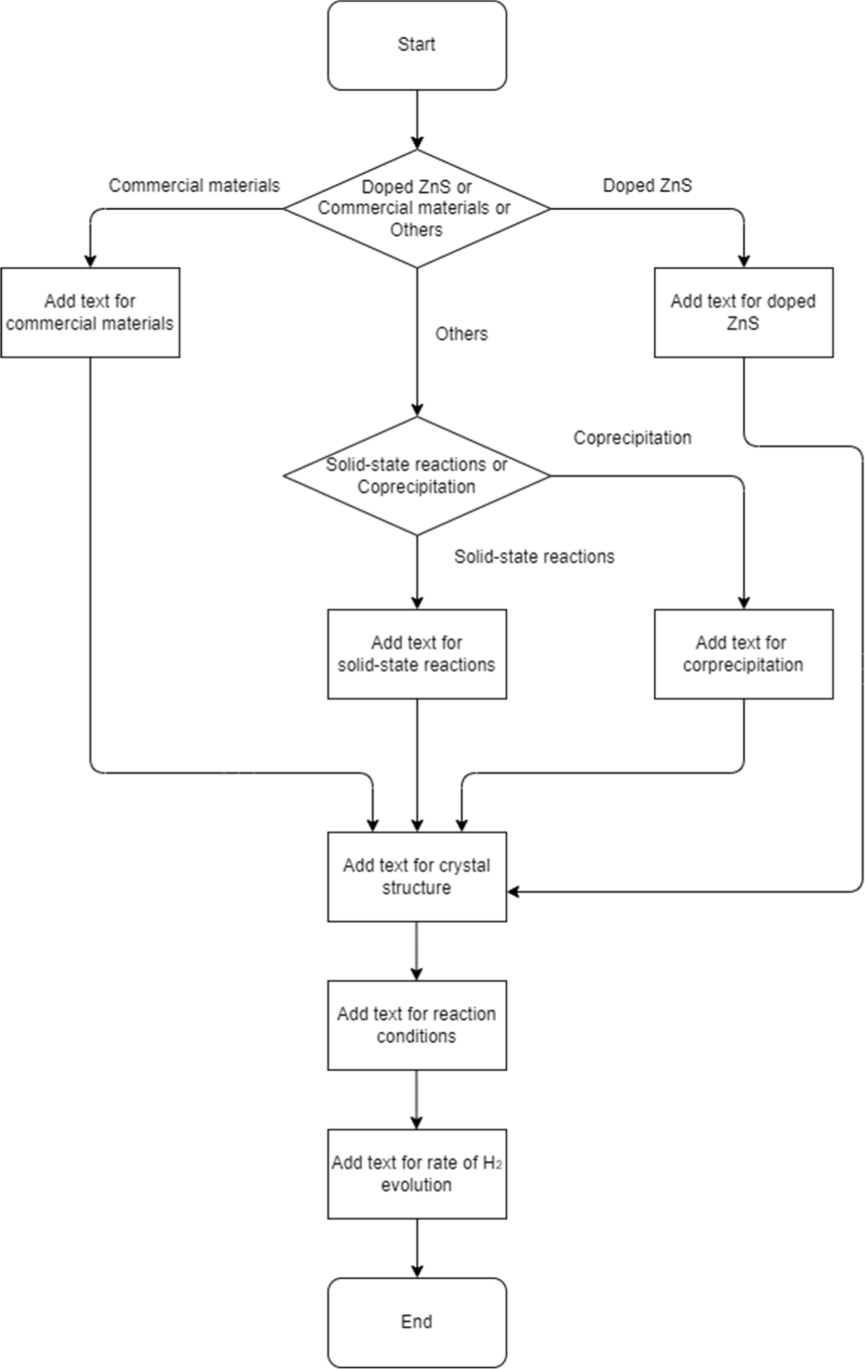
Flowchart for converting a record in an experimental CSV file into a text format using predefined sentence templates. This process enables LLMs to interpret the tabular experimental data in a contextually enriched format, making it comparable to textual information from PDF literature.

In the templates, the placeholders appear in bold within square brackets and are formatted as [parameter_name] or [parameter_name: item1/item2/···]. A placeholder whose “parameter_name” is prefixed with “type of ∼” refers to categorical attributes, whereas a placeholder with “value of ∼” refers to numerical values. When multiple values follow the colon, one value is selected based on the actual record. The conversion enables the LLMs to use tabular experimental data in a contextually enriched format. Box 2 presents typical examples of generated text.

### 2.2 LLM

Considering ease of introduction and operation for material development, as a model with fewer than 10 billion parameters that can run on a laptop, gemma-2-9b-it,^43,44^ developed by Google Inc., was employed as the main model in the present study. The LLM can be run on a local graphics processing unit (GPU) environment using an NVIDIA GeForce RTX 4090 laptop GPU with 16 GB of VRAM. The model was loaded and operated using the Hugging Face *transformers* library.^45^ In addition, 4-bit quantization was applied in the present study using the *bitsandbytes* library to reduce GPU memory requirements and shorten inference time.^46,47^

We also evaluated Qwen2.5-7B-Instruct^48,49^ as an alternative local model and gemma-2-27b-it^43,50^ as a larger local model, both with and without the framework. Qwen2.5-7B-Instruct was executed on the same laptop used for gemma-2-9b-it. Because gemma-2-27b-it has a larger parameter count and exceeded the capacity of the laptop GPU with 16 GB of VRAM, we prepared a dedicated local server equipped with an NVIDIA GeForce RTX 3090 GPU (24 GB of VRAM) to run gemma-2-27b-it. These local models were executed under the same conditions as gemma-2-9b-it. For cloud-based benchmarking, we additionally evaluated GPT-4o^51^ via the ChatGPT^7^ web interface using the same query. GPT-4o was evaluated only without the framework.

### 2.3 MDSK-RAG

Figure 3 shows the pipeline of the framework constructed to incorporate domain-specific knowledge. The pipeline contained two retrievers: one for extraction from the converted experimental records, and the other for the scientific articles. Each retriever extracted k pieces of information that were considered relevant to the input query, where k denotes the number of retrieved items.

**Figure 3:**
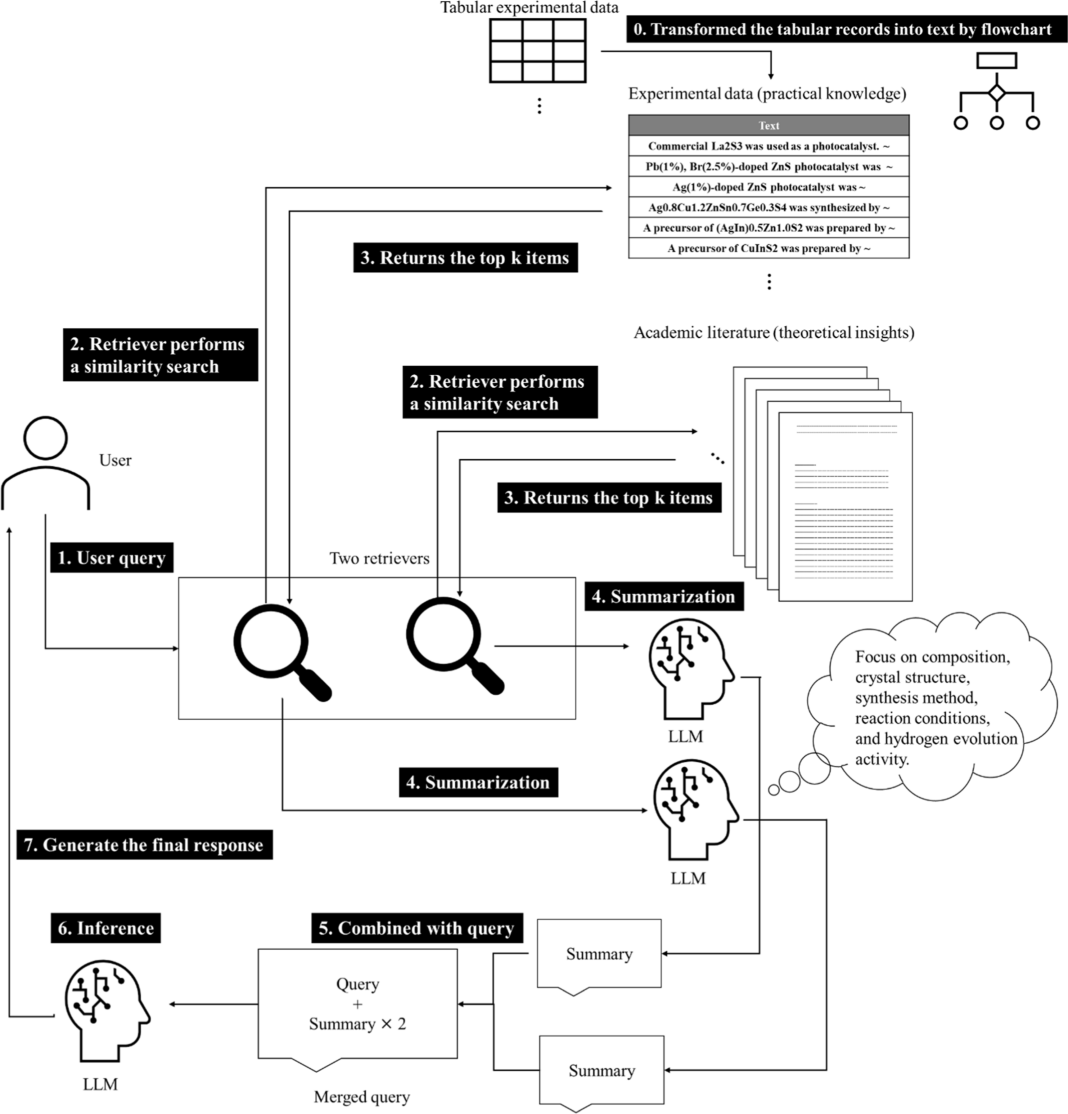
Structure of the MDSK-RAG pipeline, which combines retrieved text from experimental CSV and scientific PDFs to generate answers based on domain-specific knowledge.

To determine the retrieval size *k* used in this study, we performed a sensitivity analysis of k for the framework using the main model, gemma-2-9b-it. Considering our comparisons were paired and the sample size is small (*n* = 14), we used the Wilcoxon signed-rank test (two-sided)^52^ as a method for verifying significant differences. This choice follows precedent in the literature where the Wilcoxon test has been applied at comparable sample sizes (*n* = 12) for across-data set comparisons.^53^ We used *SciPy* library^54,55^ to apply the Wilcoxon signed-rank test. For the *n* = 14 paired comparisons, two-sided Wilcoxon signed-rank tests indicated no statistically significant difference in cosine similarity between *k* = 10 and *k* = 20 (*W* = 49.0, *p* = 0.855) nor between *k* = 10 and *k* = 5 (*W* = 32.0, *p* = 0.217). W is the Wilcoxon signed-rank test statistic and *p* is the two-sided *p*-value. Both *p*-values exceed the conventional α = 0.05 threshold; the tests did not detect a statistically significant difference between conditions (Figure S1). Average inference times reported were measured from a setup-complete state. In other words, after the model, tokenizer, and retrieval indices were loaded and a short warm-up run had been performed (Figure S2). For the 14-question evaluation, average inference time was approximately 65.1 s for *k* = 5, 73.3 s for *k* = 10, and 88.7 s for *k* = 20. This indicated that increasing k resulted in higher inference latency. We measured peak GPU VRAM and subtracted baseline (idle) usage to estimate the net VRAM required by the inference pipeline. Net VRAM increases were approximately 8.24 GB for *k* = 5, 9.30 GB for *k* = 10, and 14.75 GB for *k* = 20 (Figure S3). Although increasing k increases the amount of available context, it also raises inference latency and net GPU VRAM usage. Considering these trade-offs under our primary execution environment (a laptop with an NVIDIA GeForce RTX 4090 laptop GPU and 16 GB VRAM), we selected *k* = 10 as a pragmatic balance for operational use in this study. We note that the *k* value can be customized based on the machine specifications and intended purpose.

All the documents were vectorized using a *sentence-transformers* library^56,57^ with the all-MiniLM-L6-v2 model.^58^ The converted experimental records were read using *pandas* library,^59^ the PDF files were processed using *pypdf* library,^60^ and each source was indexed using *faiss* library^61^ for similarity searches. Subsequently, a similarity search was performed to extract the relevant information by separately comparing the vectorized input query with the vectorized content from each source.

After retrieving the ten pieces of information from the converted experimental records, these pieces were often phrased similarly for the same photocatalyst. Therefore, a summarization using the local LLM was applied to distill them into a single, concise, and representative passage. The documents were first split into chunks based on a maximum token size of 150 to enable efficient retrieval from the PDF files. This token-based chunking can result in fragmented or incomplete sentences. Therefore, to improve interpretability, the ten retrieved chunks from the PDF files were also summarized into a single coherent passage using the local LLM.

In the framework, a prompt design for summarization plays a critical role in guiding the generated passages to focus on domain-relevant information. The summary emphasizes five key aspects: composition, crystal structure type, synthesis method, reaction conditions, and hydrogen evolution activity because these factors are important for the metal-sulfide photocatalysts of the target domain as noted in the Introduction. For the retrieved information from each source, to focus on these five elements, summarization was performed using the following prompt:

“Please summarize the following text, focusing on the composition, crystal structure type, synthesis method, reaction conditions, and hydrogen evolution activity:”

Here, the local LLM was used to perform the summarization with a maximum token limit of 512 for each summary. The resulting summaries from both sources were concatenated with the input query to form a merged query. The merged query was then provided as an input query to the local LLM to generate the final answer to the question under the constraint of a maximum token limit of 512. The merged query comprises three components: (i) “Question”: followed by the input query corresponding to the expert-defined question, (ii) “Related Information”: followed by the summarized passages from the converted experimental records and PDF files, and (iii) “Answer”: which serves as a placeholder for the final response to the merged query. For clarity, an overview of the structure of the merged query is presented in Figure 4. Here, while the specification of the five keywords reflects on domain knowledge, keyword regeneration would be alternatively adopted.^62^

**Figure 4:**
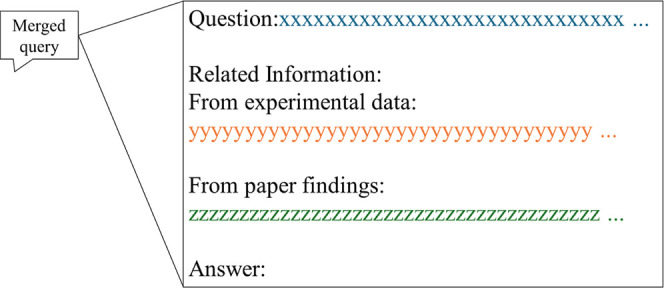
Structure of the merged query used as input to the local LLM in the MDSK-RAG framework.

### 2.4 Evaluation

The framework was evaluated using the 14 questions created by Kudo et al., who served as the domain experts. The 14 questions are presented in Box 3.

For embedding-based quantitative evaluations, the responses were vectorized into embeddings using the *sentence-transformers* library with the all-MiniLM-L6-v2 model. To evaluate how the generated text responses changed owing to the application of the framework, we visualized the vector representations of the generated responses using principal component analysis (PCA).^63^ PCA was carried out on the standardized embedding of the generated response. Next, to evaluate quantitatively how well the generated responses matched the expert-level answers, we calculated the cosine similarity between each embedding of a generated response and the embeddings of its corresponding example of answers. The examples of the answers provided by domain experts are shown in Box S1. Outputs of ChatGPT sometimes contain emojis. Prior to embedding, emojis were converted to short descriptive names using the *emoji* library.^64^

The responses generated by the framework for the main model (gemma-2-9b-it) were evaluated by another domain expert. Specifically, a five-point scale evaluation was based on comparing the responses with the framework and those without the framework.

We used the Wilcoxon signed-rank test (two-sided) as a method for verifying significant differences between generated responses. Relative percentage gain was computed by the following eq 1.

Relativepercentagegain[%]=[(medianwiththeframework−medianwithouttheframework)/(medianwithouttheframework)]×100 1

### 2.5 Web Application

An application based on the framework was implemented using Streamlit^65^ to support practical applications in daily materials development. The interface was designed to allow users to input a query, retrieve and summarize relevant information from both experimental tabular data and scientific literature, and receive a response generated by the local LLM. It aims to make the framework accessible to materials researchers without requiring programming expertise, thereby facilitating the integration of AI-driven assistance into experimental workflows.

## 3 Results and Discussion

### 3.1 Verification of MDSK-RAG Effectiveness with the Main Model

To validate the effectiveness of the framework using gemma-2-9b-it, we evaluated retrieval and summarization performance with both automated embedding-based metrics and expert human scoring. We report cosine similarity to the examples of answers, expert ratings (5-point scale), and statistical tests (Wilcoxon signed-rank test for paired comparisons). We evaluated 14 validation questions to assess the effectiveness of the framework. These questions were previously presented in Box 3. They were grouped into four categories based on their content and purpose:

- Group 1 (Q1-5): Domain-specific factual questions. These questions require knowledge of materials science.
- Group 2 (Q6, 7): Hybrid questions. These questions includes both general and domain-specific elements.
- Group 3 (Q8, 9): Reasoning-type questions. These questions requires causal explanations.
- Group 4 (Q10-14): General or abstract questions. These questions lack specific references to material systems.

Box S2 presents the output results for the 14 validation questions, comparing the responses generated with and without the framework. The responses without the framework comprised (i) the original question and (ii) the generated answer. In contrast, the response with the framework comprised the (i) original question, (ii) related information retrieved and summarized from the converted experimental records and PDF data, and (iii) generated answer. As shown in item (ii) in Box S3, two knowledge sources, namely, in-house experimental data and peer-reviewed scientific literature, were provided to the LLM in a format optimized for language model processing. This formatting is based on a carefully designed summarization prompt that directs the generated passages to focus on five domain-relevant aspects: compositions, crystal structure type, synthesis methods, reaction conditions, and hydrogen evolution activities. Without the framework, the generated responses tend to be abstract and often lack concrete and evidence-based interpretations. Even with the framework, a few responses retained abstract phrasing, although these cases were less frequent. Additionally, in the responses without the framework, a few instances were observed in which the output was truncated before completion, likely because the response exceeded the predefined token limit.

For visualization and quantitative confirmation of embedding shifts, each embedding was projected onto the first two principal components via PCA. Figure 5 shows the responses generated with and without the framework in the principal two-dimensional (2D) space constructed using PCA. Each response was plotted as a point, and paired responses to the same question were labeled with the same number. Blue circles indicate responses with the framework, whereas orange circles represent those without the framework. The spatial separation between paired responses in the principal 2D space constructed using PCA suggests that output texts differ structurally. We computed the Euclidean distance between paired PCA coordinates. In other words, the straight-line distance between each pair of responses to the same question, one produced with the framework and the other without the framework in the principal 2D space constructed using PCA. Figure 6 presents these results. Although the magnitude of the shifts varies across items, the embeddings projected onto the first two principal components (PC) exhibit systematic distance differences between the with and without the framework conditions, confirming that retrieval augmentation produces measurable shifts in the embedding representations. We note that the 2D PCA projection captures only a portion of the total variance. For all questions, the use of the framework led to responses that were structurally different from those generated without it.

**Figure 5:**
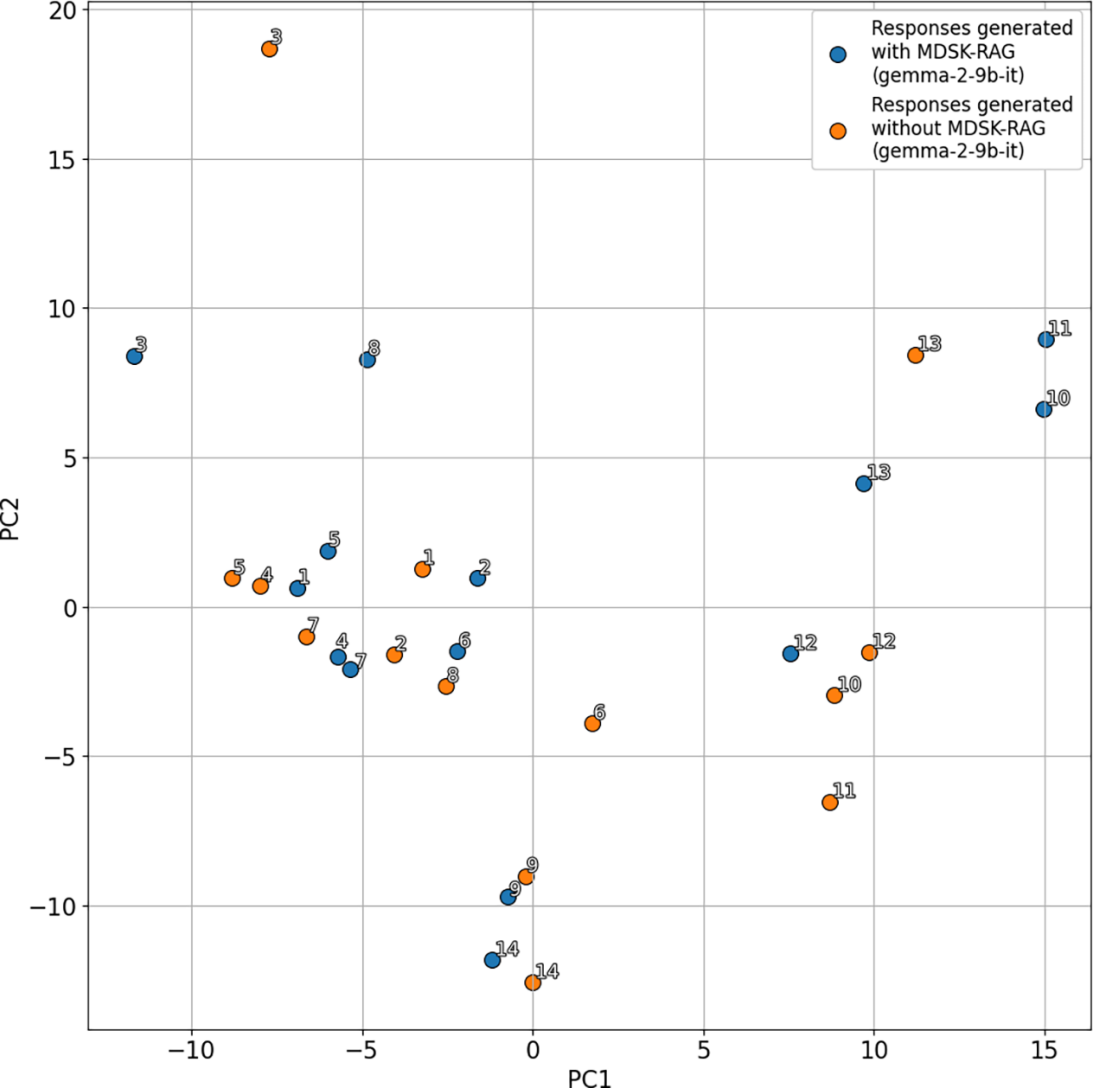
Responses generated with and without MDSK-RAG in the principal two-dimensional space constructed with PCA. Blue circles indicate responses with MDSK-RAG, and orange circles represent those without MDSK-RAG.

**Figure 6:**
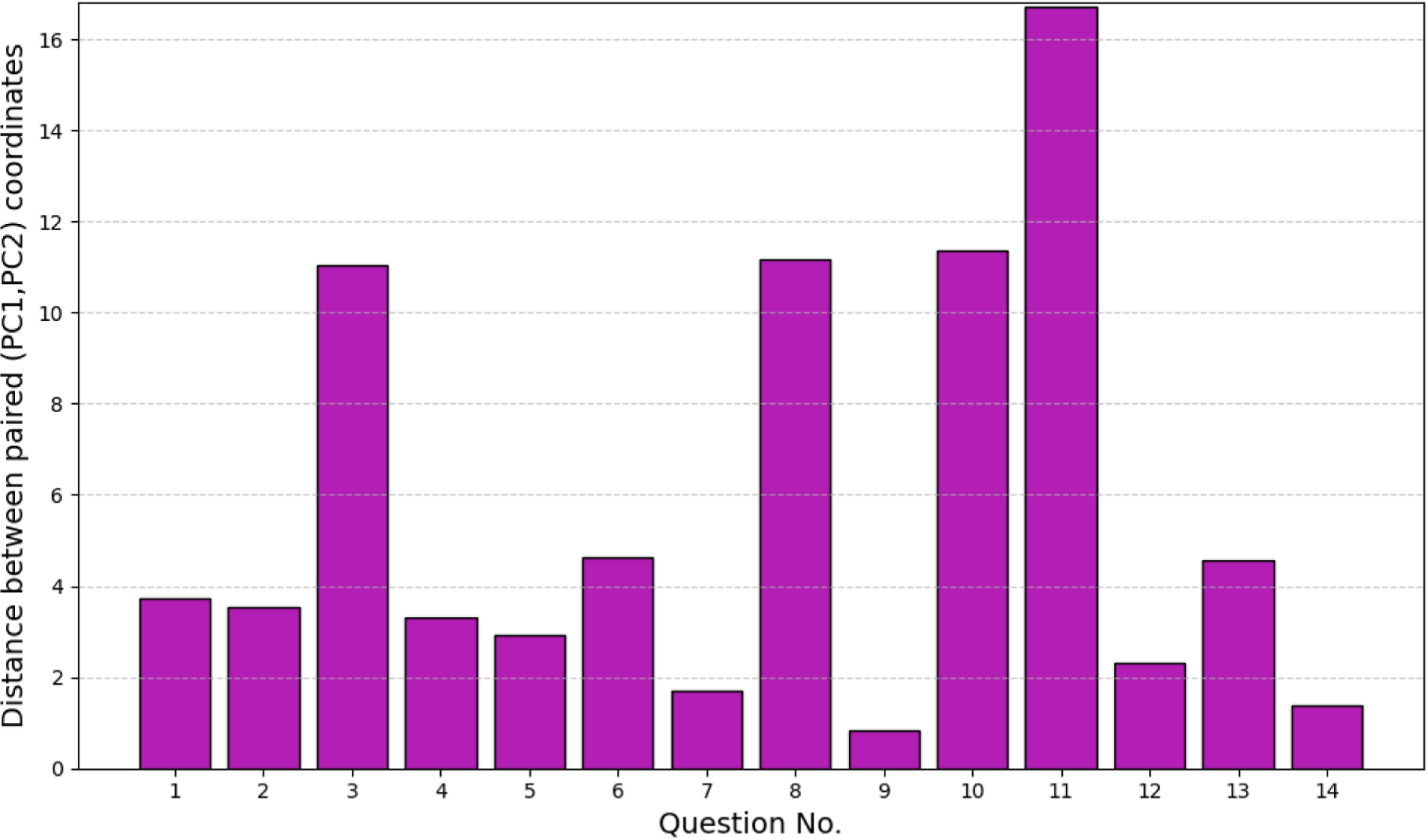
Straight-line distance between each pair of responses to the same question, one produced with the framework and the other without the framework in the principal 2D space constructed using PCA.

Figure 7 shows the cosine similarity between each of the responses (gemma-2-9b-it) generated with and without the framework and the examples of answers written by a domain expert. For the *n* = 14 paired comparisons, the Wilcoxon signed-rank test indicated a statistically significant difference in cosine similarity between responses produced with the framework and those without it (*W* = 14.0, *p* = 1.34 × 10^–2^). Thus, the null hypothesis of no median difference is rejected at the 0.05 significance level. The median cosine similarity increased from 0.63 (without the framework) to 0.71 (with the framework), an absolute increase of 0.08 corresponding to a relative percentage gain of 12.70%. The *p*-values are below the conventional α = 0.05 threshold; the tests detected a statistically significant difference between conditions. This suggests that the framework is effective in guiding the LLM to generate more domain-specific responses by anchoring outputs closer to expert-level answers.

**Figure 7:**
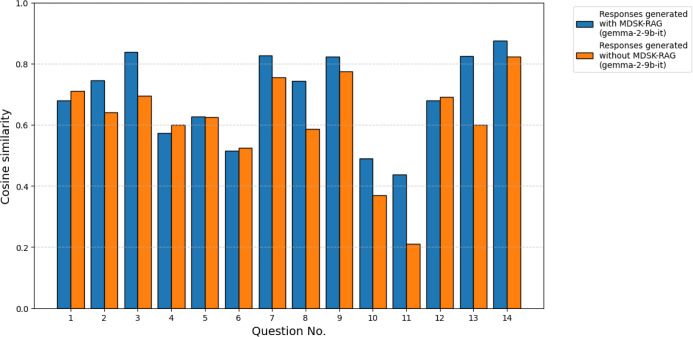
Cosine similarity between the responses (gemma-2-9b-it) and the examples of answers written by a domain expert for each question. Blue bars represent responses generated with MDSK-RAG, while orange bars represent those generated without MDSK-RAG.

Table 1 summarizes the five-point scale evaluations of the responses generated with and without the framework. A domain expert performed the evaluations. For the *n* = 14 paired items, the Wilcoxon signed-rank test indicated a statistically significant difference in expert 5-point ratings between responses produced with the framework and those without it (*W* = 3.5, *p* = 7.00 × 10^–3^). Thus, the null hypothesis of no median difference is rejected at the 0.05 significance level. The median expert 5-point rating increased from 2 (without the framework) to 3 (with the framework), an absolute increase of 1 point corresponding to a relative percentage gain of 50.00%. The *p*-values are below the conventional α = 0.05 threshold; the tests detected a statistically significant difference between conditions. These results indicate that domain experts judged outputs with the framework to be more relevant and appropriate than those without it.

**Table 1:** Five-Point Scale Evaluation of Responses Generated without and with MDSK-RAG for the 14 Expert-Defined Questions^a^

Group No.	Question No.	Without MDSK-RAG	With MDSK-RAG
1	1	2	4
	2	2	4
	3	2	4
	4	3	3
	5	2	3
2	6	2	3
	7	1	3
3	8	3	2
	9	1	3
4	10	1	2
	11	1	2
	12	1	1
	13	1	2
	14	2	2

a (A score of 1 indicates the lowest quality, while a score of 5 indicates the highest quality).

Particularly, the responses in Groups 1 and 2 with the framework received higher scores than those without the framework as shown in Table 1. For instance, Q3 asked about the synthesis strategies for a particular material. The response with the framework included concrete experimental conditions (Box S2). These details were obtained from the experimental tabular data (Box S3: From experimental data). In addition, the responses included insights from published literature (Box S3: From paper findings). The answers were both specific and supported by evidence. In contrast, responses without the framework lacked these details and remained abstract.

In Group 3, both an increase (Q9) and a decrease (Q8) in scores with the framework were observed. The response for Q8 with the framework included signs that the model attempted reasoning based on the retrieved information and it contained several misunderstandings (Box S2). The score for the response with the framework was 1 point lower than that of the response without the framework as shown in Table 1. By contrast, the response for Q9 contained evidence provided with appropriate context (“The text you provided highlights several reasons why employing a metal sulfide photocatalyst as a single particle for water splitting is difficult”) (Box S2). The score for the response with it was 2 points higher than that of the response without it as shown in Table 1. A likely explanation is the different nature of evidence in the knowledge sources. For Q9, reviews and other papers include clear evaluative statements. MDSK-RAG can retrieve those statements and reproduce them in its answers. For Q8, they rarely give direct conclusions about how solid-solution formation affects photocatalytic activity. Instead, they include separate observations, such as changes in band structures or absorption properties due to formation of solid-solution. The framework would struggle to combine these separate observations into one accurate conclusion. This difficulty explains the incorrect reasoning seen in the Q8 responses. This explanation is consistent with the observation that the response for Q9 contained evidence provided with appropriate context. These results suggest that LLMs may attempt partial reasoning even when the retrieved context is incomplete; insufficient contextual information can lead to confusion in the reasoning process and to incorrect conclusions.

In Group 4, the benefit of the framework was limited compared with that in Groups 1 and 2. However, the responses generated with the framework still exhibited an overall trend of improved response quality compared with those without the framework as shown in Table 1.

The evaluations highlighted the limitation of relying solely on the retrieved content that matches the query. To address reasoning-based or abstract tasks effectively, future work should explore frameworks that can provide all the necessary contexts required to solve a given task, instead of only partial matches from retrieval. As a specific future direction, a hybrid symbolic approach could be considered. For example, constructing a knowledge store specialized for metal-sulfide photocatalysts could enable checking LLM outputs for inconsistencies or missing prerequisites. Such an approach may also contribute to improved performance in tasks involving more general or abstract matters.

For the majority of the 14 questions, the results demonstrate that the responses generated with the framework received higher scores than those without the framework. These results indicate that the integration of external, domain-specific knowledge through the framework not only enhances factual accuracy but also improves the domain specificity of the response, and that the specificity of the questions plays an important role. For general or abstractly phrased questions, the LLM tends to generate vague responses, even with the framework. In contrast, questions that include specific terminology or proper nouns, such as AgGaS_2_ in Q3, enable the model to generate more accurate and relevant answers. Overall, the five-point scale evaluation supports the fact that the framework improves the quality of responses generated in most cases, particularly when questions are well-defined and contextualized. These findings demonstrate that the framework effectively enhances both the factual accuracy and domain specificity of responses from a local LLM in the context of metal-sulfide photocatalyst research.

### 3.2 Effect of Local LLM Choice

To verify the effect of local LLM choice, we compared outputs with and without the framework using Qwen2.5-7B-Instruct. Figure 8 shows the cosine similarity between each of the responses (Qwen2.5-7B-Instruct) generated with and without the framework and the examples of answers written by a domain expert. For the *n* = 14 paired comparisons, a Wilcoxon signed-rank test revealed a statistically significant difference in cosine similarity between responses generated with the framework and those generated without the framework (*W* = 14.0, *p* = 1.34 × 10^–2^). The median cosine similarity increased from 0.64 (without the framework) to 0.71 (with the framework), an absolute increase of 0.07 corresponding to a relative percentage gain of 10.94%. The *p*-values are below the conventional α = 0.05 threshold; the tests detected a statistically significant difference between conditions. These results indicate that, for Qwen2.5-7B-Instruct and the 14-question evaluation set, the framework produced responses that were consistently more similar to the expert references. This analysis shows that the approach also yields consistent benefits with another local LLM.

**Figure 8:**
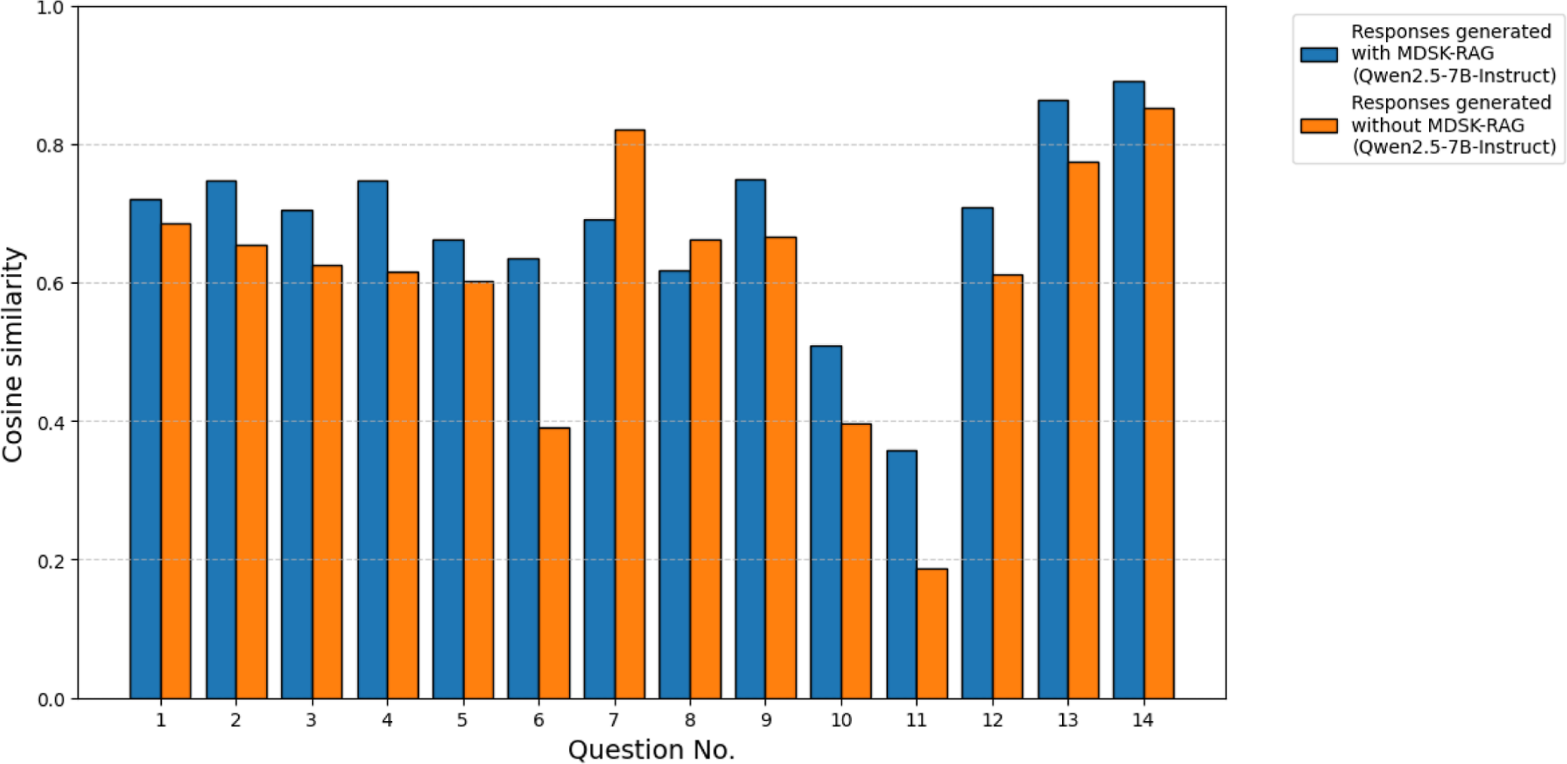
Cosine similarity between the responses (Qwen2.5-7B-Instruct) and the examples of answers written by a domain expert for each question. Blue bars represent responses generated with MDSK-RAG, while orange bars represent those generated without MDSK-RAG.

### 3.3 Scalability to a Larger LLM

To verify the scalability to a larger LLM, we compared outputs with and without the framework using gemma-2-27b-it. Figure 9 shows the cosine similarity between each of the responses (gemma-2-27b-it) generated with and without the framework and the examples of answers written by a domain expert. For the *n* = 14 paired comparisons, a Wilcoxon signed-rank test revealed a statistically significant difference in cosine similarity between responses generated with the framework and those generated without the framework (*W* = 0.0, *p* = 1.22 × 10^–4^). The median cosine similarity increased from 0.67 (without the framework) to 0.73 (with the framework), an absolute increase of 0.06 corresponding to a relative percentage gain of 8.96%. The *p*-values are below the conventional α = 0.05 threshold; the tests detected a statistically significant difference between conditions. These results indicate that, for gemma-2-27b-it and the 14-question evaluation set, the framework produced responses that were consistently more similar to the expert references. While our main experiments used a local model within the <10 billion parameters (gemma-2-9b-it), we found that the MDSK-RAG approach also yields consistent benefits with larger local models.

**Figure 9:**
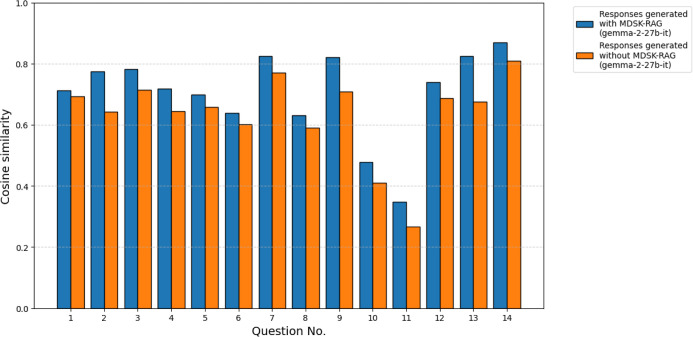
Cosine similarity between the responses (gemma-2-27b-it) and the examples of answers written by a domain expert for each question. Blue bars represent responses generated with MDSK-RAG, while orange bars represent those generated without MDSK-RAG.

### 3.4 Comparison with Cloud-Based LLM

To compare performance against a cloud-based LLM, we compared responses produced by gemma-2-9b-it with the framework and those produced by GPT-4o without the framework. For example, seeing Q3 as a representative case, the response produced by GPT-4o without the framework lacked the concrete experimental details that appeared in the response produced by gemma-2-9b-it with the framework and remained comparatively abstract (Box S4). Figure 10 shows the cosine similarity between each response (gemma-2-9b-it with the framework and GPT-4o without the framework) and the examples of answers written by a domain expert. For the *n* = 14 paired comparisons, the Wilcoxon signed-rank test indicated a statistically significant difference in cosine similarity between responses produced by gemma-2-9b-it with the framework and those produced by GPT-4o without the framework (*W* = 20.0, *p* = 4.20 × 10^–2^). The median cosine similarity was 0.71 for responses produced by gemma-2-9b-it with the framework and 0.66 for responses produced by GPT-4o without the framework. The *p*-values are below the conventional α = 0.05 threshold; the tests detected a statistically significant difference between conditions. Although GPT-4o is a high-performance cloud-based model, these results suggest that a locally deployed model with fewer than 10 billion parameters, when domain-specialized via the framework, can in certain domain-specific tasks generate responses that more closely match expert reference answers than those from a higher-capability cloud model under the present task design. Our framework is designed to solve domain-specific tasks, while general cloud models tend to provide generic answers and are not necessarily superior for such domain-specific tasks.

**Figure 10:**
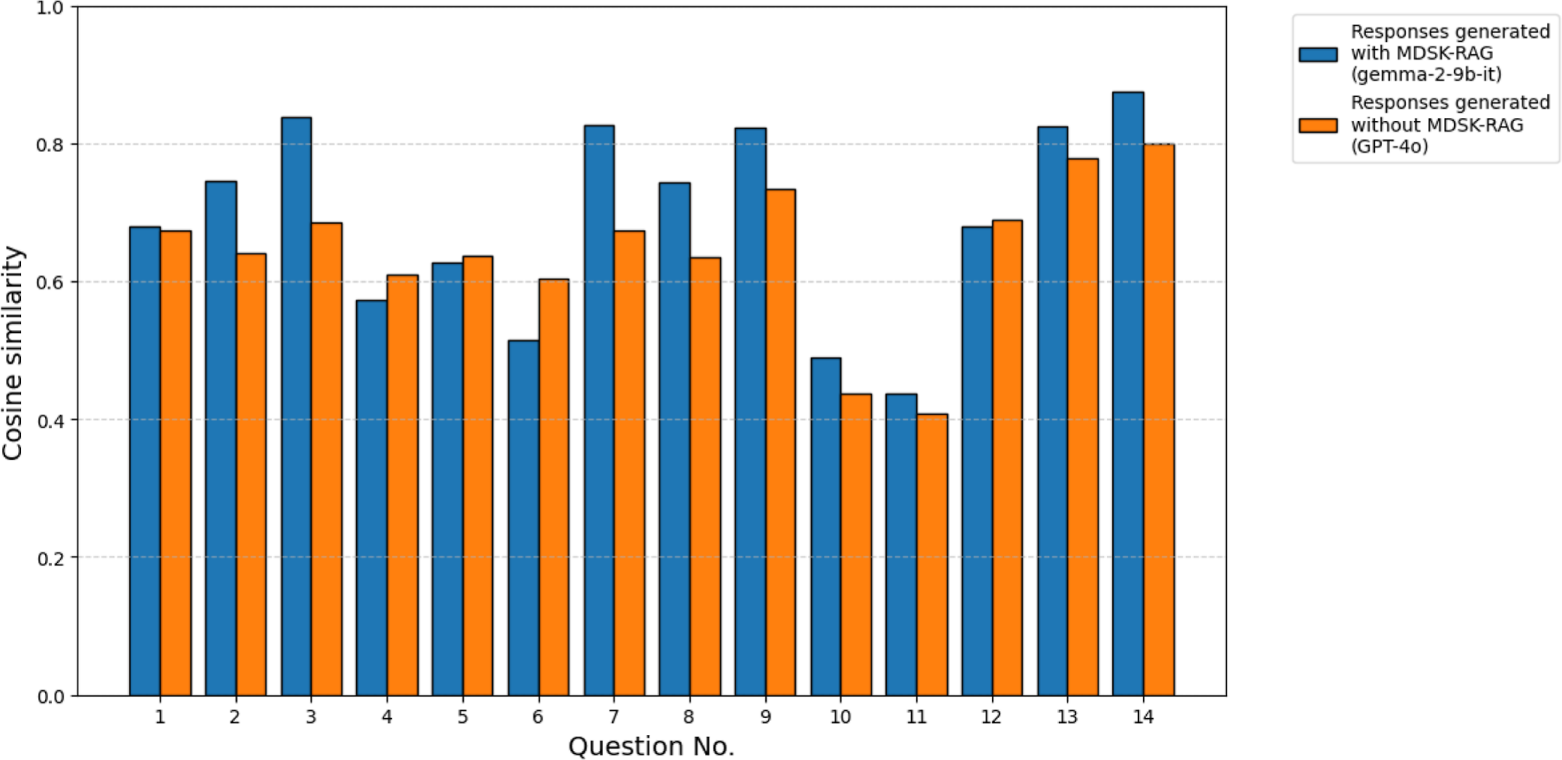
Cosine similarity between the responses and the examples of answers written by a domain expert for each question. Blue bars represent responses (gemma-2-9b-it) generated with MDSK-RAG, while orange bars represent those (GPT-4o) generated without MDSK-RAG.

### 3.5 Web Application

Figure 11 shows the web application interface developed in this study using Streamlit. This interface implements the framework. Users can type a question into the “Enter your question” field and then click the “Get related information and answer” button to receive the results. The output is presented in a structured format with three sections: question, related information, and answer. The related information section contains the retrieved and summarized content from two sources: the converted experimental records and scientific literature in PDF format. These sources provide a context for the local LLM. The LLM uses this contextual information to generate the final answer. In addition, each ranked list of top-k items by similarity search is displayed after the three sections to allow users to inspect the specific evidence items used for answering. The display of the top-k retrieved items is omitted. The interface was developed to be practical for materials researchers. This enables users to access the system without programming skills. This design supports the integration of AI-driven assistance into everyday decision making in experimental materials research.

**Figure 11:**
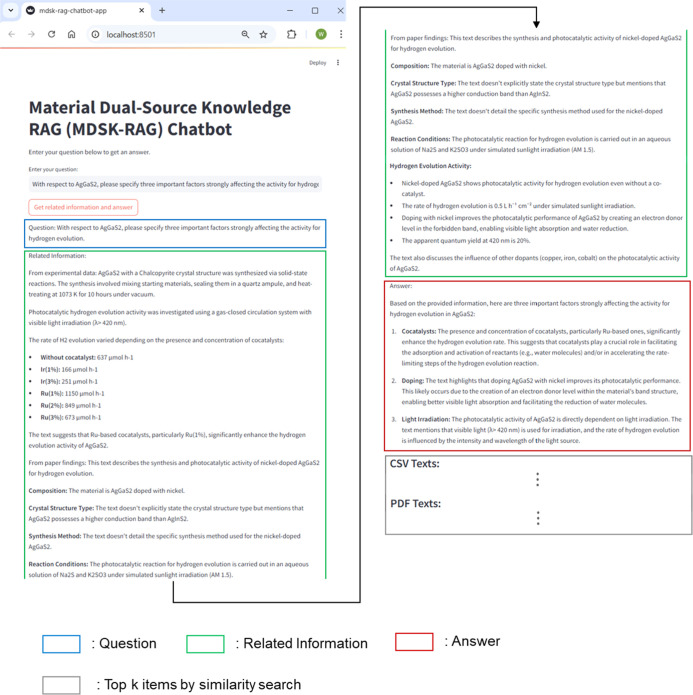
Example of the application’s graphical user interface for querying the MDSK-RAG framework. Black arrows indicate that the page continues beyond the visible region.

### 3.6 Discussion

These results should be interpreted considering the specific task setup used in the present study. Although we used metal sulfide photocatalysts as a case study, extending the framework to other material classes will require materials-specific refinement. Such refinement includes selection and curation of source documents, both CSV and PDF, that contain relevant experimental and tabular data for the target class. It also requires the design of flowcharts and conversion templates that reliably transform tabular CSV files into text representations the model can consume, and these should be able to comprehensively capture the characteristic features of the target material system. In addition, an explicit process is needed to enumerate the material attributes and performance metrics that should be emphasized for each domain and to incorporate those elements into the summary prompts used by the system. The considerations and workflows presented in this paper can serve as a practical reference and starting point for researchers seeking to adapt the framework to other material classes.

## 4 Conclusions

We developed MDSK-RAG, a RAG framework that adapts a locally hosted large language model to materials research by unifying structured in-house experimental records and peer-reviewed literature without fine-tuning models. Using metal-sulfide photocatalysts as a case study, we built a knowledge base of 740 experimental records and 20 scientific PDFs and implemented the pipeline primarily with a quantized gemma-2-9b-it model running locally. On a benchmark consisting of 14 expert-defined questions, the framework improved alignment with expert answers: for gemma-2-9b-it, the median cosine similarity increased from 0.63 to 0.71, an absolute increase of 0.08 (corresponding to a relative percentage gain of 12.70%; Wilcoxon signed-rank test: *W* = 14.0, *p* = 1.34 × 10^–2^) and the median expert 5-point rating rose from 2 to 3, an absolute increase of 1 point (corresponding to a relative percentage gain of 50.00%; Wilcoxon signed-rank test: *W* = 3.5, *p* = 7.0 × 10^–3^). Comparable and statistically significant gains were observed for Qwen2.5-7B-Instruct and for gemma-2-27b-it when evaluated by cosine similarity of sentence embeddings, indicating that the framework enhancement is effective across different local model architectures and that its benefits persist as model size increases. In a cloud comparison, gemma-2-9b-it augmented with the framework achieved the median cosine similarity of 0.71 while GPT-4o produced the median cosine similarity of 0.66 (Wilcoxon signed-rank test: *W* = 20, *p* = 4.20 × 10^–2^). These results suggest that even local LLMs with fewer than 10 billion parameters, when domain-specialized using the framework, can in certain domains produce responses that more closely match expert reference answers than those generated by high-performance cloud-based models. These results demonstrate that the framework is a practical tool for laboratory environments that handle proprietary or unpublished records. At the same time, we observed failure modes: reasoning-type queries sometimes produced lower expert scores after augmentation, consistent with partial or incomplete retrieved context provoking erroneous model inferences. To mitigate this risk, future work will explore hybrid symbolic approaches, for example by constructing a domain-specific knowledge store for metal-sulfide photocatalysts to enable consistency checks and so on. Such enhancements may also improve the framework’s robustness and applicability to more general or abstract tasks. In summary, the framework provides a practical and extensible route to adapt local LLMs for materials-science tasks in privacy-sensitive laboratory environments. By fusing in-house experimental records with the scientific literature in a retrieval-driven manner and without model retraining, the framework improves the domain specificity and expert alignment of generated responses and offers a promising tool to augment decision making and accelerate iterative experiment cycles in materials research. We note that the framework demonstrated for metal-sulfide photocatalysts is effective but requires material-specific refinement to be applied to other material systems. The framework demonstrates a feasible path for LLM assistance to materials research under local, privacy-sensitive constraints.

## Appendix

### Box 1

Predefined sentence templates used for converting tabular experimental data into text-based format. Each template includes placeholders for experimental parameters and is applied at a corresponding step in the conversion flowchart shown in Figure 2 to produce interpretable text-based inputs for the MDSK-RAG pipeline.

**< Sentence template for “Add text for commercial materials”>**

Commercial [**type of Host**] was used as a photocatalyst.

**< Sentence template for “Add text for doped ZnS”>**

(Vacuum)

[**type of dopA_element**] ([**value of dopA_element**]%), [**dopB_element**] ([**value of dopB_element**]%)-doped ZnS photocatalyst was prepared by mixing an aqueous solution containing Zn, [**type of dopA_element**], [**type of dopB_element**]. The obtained precipitates were washed with distilled water using a centrifuge and then were sealed in a quartz ampule tube in vacuo and heat-treated at [**value of Calcination_Temp_Stage1**] for [**value of Calcination_Time_Stage1**] h.

(N_2_ flow)

[**type of dopA_element**] ([**value of dopA_element**]%), [**dopB_element**] ([**value of dopB_element**]%)-doped ZnS photocatalyst was prepared by mixing an aqueous solution containing Zn, [**type of dopA_element**], [**type of dopB_element**]. The obtained precipitates were washed with distilled water using a centrifuge and then heat-treated at [**value of Calcination_Temp_Stage1**] for [**value of Calcination_Time_Stage1**] h in a N_2_ flow.

**< Sentence template for “Add text for solid-state reactions”>**

(with excess amounts)

[**type of Host**] was synthesized by solid-state reactions. The starting materials were mixed with excess amounts of [**type of excess2_element**] with [**value of excess2_element**]%, [**type of excess2_element**] with [**value of excess2_element**]%, ... , [**type of excess2_element**] with [**value of excess2_element**]%. The mixture was sealed in a quartz ampule tube in vacuo and heat-treated at [**value of Calcination_Temp_Stage1**] for [**value of Calcination_Time_Stage1**].

(without excess amounts)

[**type of Host**] was synthesized by solid-state reactions. The starting materials were mixed and then sealed in a quartz ampule tube in vacuo and heat-treated at [**value of Calcination_Temp_Stage1**] for [**value of Calcination_Time_Stage1**].

**< Sentence template for “Add text for coprecipitation”>**

(Vacuum)

A precursor of [**type of Host**] was prepared by the following method. An aqueous solution containing starting materials with excess amounts of [**type of excess2_element(1)**] with [**value of excess2_element(1)**]%, [**type of excess2_element(2)**] with [**value of excess2_element(2)**]%, ... , [**type of excess2_element­(n)**] with [**value of excess2_element­(n)**]% was purged with N_2_ gas. A precursor of [**type of Host**] was precipitated and then sealed in a quartz ampule tube in vacuo and heat-treated at [**value of Calcination_Temp_Stage1**] for [**value of Calcination_Time_Stage1**] h.

(N_2_ flow)

A precursor of [**type of Host**] was prepared by the following method. An aqueous solution containing starting materials with excess amounts of [**type of excess2_element(1)**] with [**value of excess2_element(1)**]%, [**type of excess2_element(2)**] with [**value of excess2_element(2)**]%, ... , [**type of excess2_element­(n)**] with [**value of excess2_element­(n)**]% was purged with N_2_ gas. A precursor of [**type of Host**] was precipitated and then heat-treated at [**value of Calcination_Temp_Stage1**] for [**value of Calcination_Time_Stage1**] h in a N_2_ flow.

**< Sentence template for “Add text for crystal structure”>**

The crystal structure of [**type of Host**] was the [**type ofcrystal structure**] structure.

**< Sentence template for “Add text for reaction conditions”>**

Photocatalytic reactions were conducted in a gas-closed circulation system with a [**type of irradiation: side/top)**] window. The photocatalyst powder ([**value of catalyst_mass**] g) was dispersed in [**type of sacrificial agent: a mixed aqueous solution of Na_2_S and K_2_SO_3_/an aqueous solution of K_2_SO_3_**]. The photocatalysts were irradiated with visible light [**type of bandpass filter: (λ = 420 nm) through bandpass filter/(λ > 420 nm) through a cutoff filter**] from a 300 W Xe lamp. [**type of cocat_element**] ([**value of cocat_element**]%) cocatalyst was loaded by [**type of loading method: a photodeposition/an impregnation/an impregnation-H_2_reduction/a predeposition/an ex situ photodeposition/an ex situ photodeposition-Heating**] method.

**< Sentence template for “Add text for rate of H_2_ evolution”>**

(For non-doped photocatalyst with the rate of H_2_ evolution)

The rate of H_2_ evolution on the “**type of Host**” was “**value of H_2_**” μmol h^–1^.

(For doped photocatalyst with the rate of H_2_ evolution)

The rate of H_2_ evolution on the [**type of dopA_element**] ([**value of dopA_element**]%), [**type of dopB_element**] ([**value of dopB_element**]%)-doped ZnS photocatalyst was [**value of H_2_**] μmol h^–1^.

(For non-doped photocatalyst with no the rate of H_2_ evolution)

[**type of Host**] did not show the activity for H_2_ evolution.

(For doped photocatalyst with no the rate of H_2_ evolution)

[**type of dopA_element**] ([**value of dopA_element**]%), [**dopB_element**] ([**value of dopB_element**]%)-doped ZnS photocatalyst did not show the activity for H_2_ evolution.

### Box 2

Typical examples of the text generated through the application of the flowchart and sentence templates. These examples demonstrate how tabular data are converted into text-based input for the MDSK-RAG pipeline, as outlined in Figure 2. The conversion process follows a specific path in the flowchart for each example, and the corresponding path is indicated in the table.

**Path in the flowchart: “Add text for commercial materials” → “Add text for crystal structure” → “Add text for reaction conditions” → “Add text for rate of H_2_ evolution.”**

Generated text: Commercial La_2_S_3_ was used as a photocatalyst. The crystal structure of La_2_S_3_ was the La_2_S_3_ structure. Photocatalytic reactions were conducted in a gas-closed circulation system with a top window Pyrex cell. The photocatalyst powder (0.3 g) was dispersed in an aqueous solution of K_2_SO_3_. The photocatalysts were irradiated with visible light (λ > 420 nm) through a cutoff filter from a 300 W Xe lamp. The rate of H_2_ evolution on the La_2_S_3_ photocatalyst was 2.4 μmol h^–1^.

**Path in the flowchart: “Add text for doped ZnS” (Vacuum) → “Add text for crystal structure” → “Add text for reaction conditions” → “Add text for rate of H_2_ evolution.”**

Generated text: Ag(1%)-doped ZnS photocatalyst was prepared by mixing an aqueous solution containing Zn, Ag. The obtained precipitates were washed with distilled water using a centrifuge and then were sealed in a quartz ampule tube in vacuo and heat-treated at 773 K for 2 h. The crystal structure of ZnS was the Zinc blende structure. Photocatalytic reactions were conducted in a gas-closed circulation system with a top window Pyrex cell. The photocatalyst powder (0.3 g) was dispersed in an aqueous solution of K_2_SO_3_. The photocatalysts were irradiated with visible light (λ > 420 nm) through a cutoff filter from a 300 W Xe lamp. The rate of H_2_ evolution on the Ag(1%)-doped ZnS photocatalyst was 8 μmol h^–1^.

**Path in the flowchart: “Add text for doped ZnS” (N_2_ flow) → “Add text for crystal structure” → “Add text for reaction conditions” → “Add text for rate of H_2_ evolution.”**

Generated text: Pb(1%), Br(2.5%)-doped ZnS photocatalyst was prepared by mixing an aqueous solution containing Zn, Pb, Br. The obtained precipitates were washed with distilled water using a centrifuge and then were heat-treated at 773 K for 2 h in a N_2_ flow. The crystal structure of ZnS was the Zinc blende structure. Photocatalytic reactions were conducted in a gas-closed circulation system with a top window Pyrex cell. The photocatalyst powder (0.3 g) was dispersed in an aqueous solution of K_2_SO_3_. The photocatalysts were irradiated with visible light (λ > 420 nm) through a cutoff filter from a 300 W Xe lamp. The rate of H_2_ evolution on the Pb(1%), Br(2.5%)-doped ZnS photocatalyst was 31 μmol h^–1^.

**Path in the flowchart: “Add text for solid-state reactions” → “Add text for crystal structure” → “Add text for reaction conditions” → “Add text for rate of H_2_ evolution.”**

Generated text: Ag_0.8_Cu_1.2_ZnSn_0.7_Ge_0.3_S_4_ was synthesized by solid-state reactions. The starting materials were mixed with excess amounts of Ge with 15%, Zn with 15%, Sn with 5%. The mixture was sealed in a quartz ampule tube in vacuo and heat-treated at 923 K for 10 h. The crystal structure of Ag_0.8_Cu_1.2_ZnSn_0.7_Ge_0.3_S_4_ was the Stannite structure. Photocatalytic reactions were conducted in a gas-closed circulation system with a top window Pyrex cell. The photocatalyst powder (0.3 g) was dispersed in a mixed aqueous solution of Na_2_S and K_2_SO_3_. The photocatalysts were irradiated with visible light (λ > 420 nm) through a cutoff filter from a 300 W Xe lamp. Ru(0.5%) cocatalyst was loaded by a photodeposition method. The rate of H_2_ evolution on the Ru(0.5%)-loaded Ag_0.8_Cu_1.2_ZnSn_0.7_Ge_0.3_S_4_ photocatalyst was 334 μmol h^–1^.

**Path in the flowchart: “Add text for coprecipitation” (Vacuum) → “Add text for crystal structure” → “Add text for reaction conditions” → “Add text for rate of H_2_ evolution.”**

Generated text: A precursor of CuInS_2_ was prepared by the following method. An aqueous solution containing starting materials with an excess amount of In with 10% was purged with N_2_ gas. A precursor of CuInS_2_ was precipitated and then sealed in a quartz ampule tube in vacuo and heat-treated at 1173 K for 5 h. The crystal structure of CuInS_2_ was the Chalcopyrite structure. Photocatalytic reactions were conducted in a gas-closed circulation system with a top window Pyrex cell. The photocatalyst powder (0.3 g) was dispersed in a mixed aqueous solution of Na_2_S and K_2_SO_3_. The photocatalysts were irradiated with visible light (λ > 420 nm) through a cutoff filter from a 300 W Xe lamp. Ru(1%) cocatalyst was loaded by a photodeposition method. The rate of H_2_ evolution on the Ru(1%)-loaded CuInS_2_ photocatalyst was 148 μmol h^–1^.

**Path in the flowchart: “Add text for coprecipitation” (N_2_ flow) → “Add text for crystal structure” → “Add text for reaction conditions” → “Add text for rate of H_2_ evolution.”**

Generated text: A precursor of (AgIn)_0.5_Zn_1.0_S_2_ was prepared by the following method. An aqueous solution containing starting materials was purged with N2 gas. A precursor of (AgIn)_0.5_Zn_1.0_S_2_ was precipitated and then heat-treated at 1123 K for 5 h in a N_2_ flow. The crystal structure of (AgIn)_0.5_Zn_1.0_S_2_ was the Wurtzite structure. Photocatalytic reactions were conducted in a gas-closed circulation system with a top window Pyrex cell. The photocatalyst powder (0.3 g) was dispersed in an aqueous solution of K_2_SO_3_. The photocatalysts were irradiated with visible light (λ > 420 nm) through a cutoff filter from a 300 W Xe lamp. The rate of H_2_ evolution on the (AgIn)_0.5_Zn_1.0_S_2_ photocatalyst was 5 μmol h^–1^.

### Box 3

Fourteen expert-defined questions used for evaluating the MDSK-RAG framework.

**Question 1**

For metal sulfide photocatalysts containing Ag as a constituent element, please specify three important factors strongly affecting the activity for hydrogen evolution.

**Question 2**

For metal sulfide photocatalysts containing Cu as a constituent element, please specify three important factors strongly affecting the activity for hydrogen evolution.

**Question 3**

With respect to AgGaS2, please specify three important factors strongly affecting the activity for hydrogen evolution.

**Question 4**

For metal sulfide photocatalysts with a chalcopyrite structure, please specify three important factors strongly affecting the activity for hydrogen evolution.

**Question 5**

For metal sulfide photocatalysts with a stannite structure, please specify three important factors strongly affecting the activity for hydrogen evolution.

**Question 6**

Please enumerate five crystal structures of metal sulfide photocatalysts with a trend of the high activity for hydrogen evolution.

**Question 7**

Please enumerate three cocatalysts of metal sulfide photocatalysts with a trend of the high activity for hydrogen evolution.

**Question 8**

For metal sulfide photocatalysts, please explain the mechanism of which formation of solid-solution affects the activity for hydrogen evolution.

**Question 9**

Please explain the reason why it is difficult to employ a metal sulfide photocatalyst as a single particle for water splitting.

**Question 10**

Please specify several examples of elements constituting the valence band of metal sulfide photocatalysts.

**Question 11**

Please specify several examples of elements constituting the conduction band of metal sulfide photocatalysts.

**Question 12**

Please specify metal sulfide photocatalysts with response to long wavelength of visible light up to 600 nm and explain the reason why those photocatalysts can response to long wavelength of visible light in terms of their band structures.

**Question 13**

For metal sulfide photocatalysts, please specify common features of elements constituting valence band and conduction band, respectively.

**Question 14**

Please specify the reason why development of metal sulfide photocatalysts with respond to long wavelength of visible light is important for practical solar water splitting.

## Supplementary Material



## Data Availability

The python code developed in this study and the experimental data samples (selected csv) are available in the following repository: https://github.com/mipypf/mdsk-rag.

## Notes

Comparison of output results varying the *k* value (Figure S1); comparison of inference time varying the *k* value (Figure S2); comparison of GPU usage varying the *k* value (Figure S3); example of answers for the 14 expert-defined questions (Box S1); comparison of the output responses generated with and without MDSK-RAG for the 14 expert-defined questions (Box S2); related information retrieved and summarized from the CSV and PDF data via MDSK-RAG for the 14 expert-defined questions (Box S3); and public URLs of output responses generated using GPT-4o for the 14 expert-defined questions (Box S4) (PDF)
