# Supplementary material for: Materials Dual-Source Knowledge Retrieval-Augmented Generation for Local Large Language Models in Photocatalysts
Source: J Chem Inf Model. 2025 Dec 1;65(24):13098–114. doi: 10.1021/acs.jcim.5c01941 (PMC12728951; doi:10.1021/acs.jcim.5c01941)
Supplement: Supplementary file 1 [file ci5c01941_si_001.pdf]

Supporting Information

Materials Dual-Source Knowledge Retrieval-Augmented Generation for Local Large Language Models in Photocatalysts

Wataru Takahara[a], Yuichi Yamaguchi[b,c*], Mai Ogano[b], Fuga Kakami[b], Yosuke Harashima[a,d*], Tomoaki Takayama[a,d], Shogo Takasuka[a], Akihiko Kudo[b,c*] and Mikiya Fujii[a,d,e*]

[a]*Division of Materials Science, Nara Institute of Science and Technology, Ikoma-shi, Nara-ken, 630-0192, Japan*

[b]*Department of Applied Chemistry, Faculty of Science, Tokyo University of Science, Shinjuku-ku, Tokyo, 162-8601, Japan*

[c]*Carbon Value Research Center, Research Institute for Science & Technology, Tokyo University of Science, Noda-shi, Chiba-ken 278-8510, Japan*

[d]*Data Science Center, Nara Institute of Science and Technology, Ikoma-shi, Nara-ken, 630-0192, Japan*

[e]*Center for Material Research Platform, Nara Institute of Science and Technology, Ikoma-shi, Nara-ken, 630-0192, Japan*

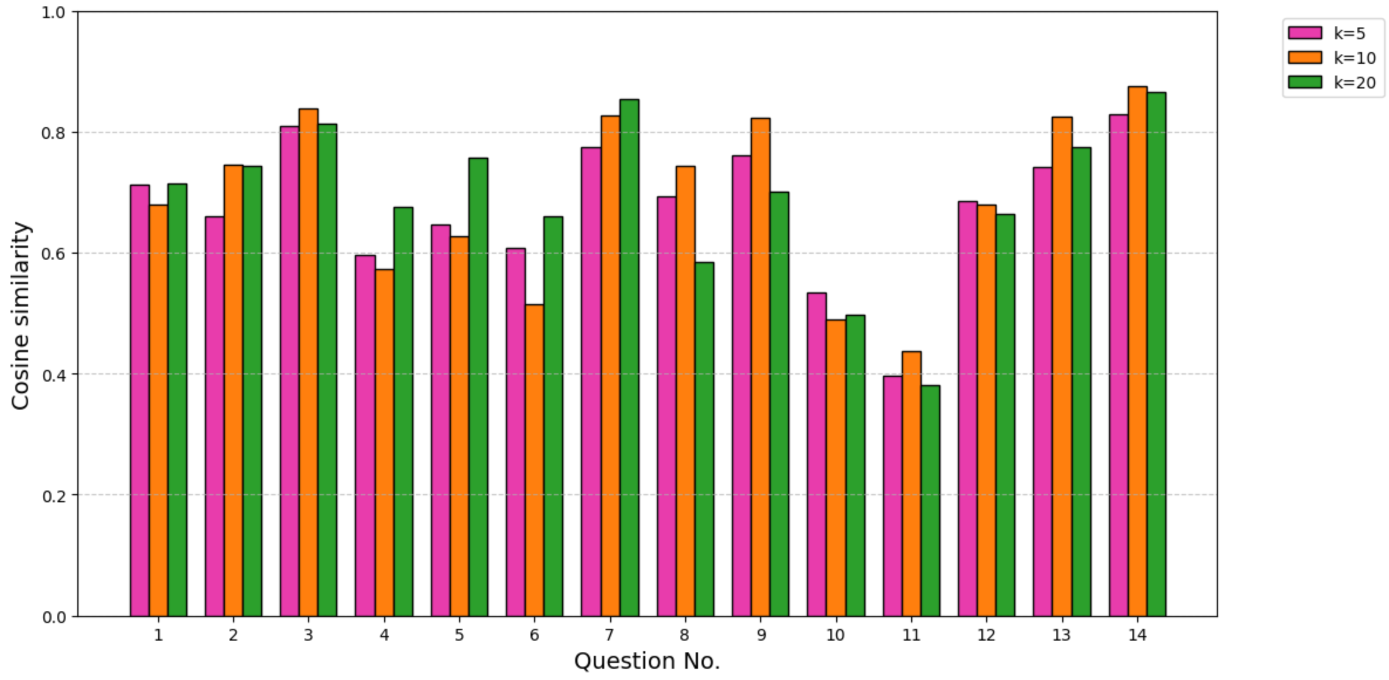

Figure S1 Cosine similarity between the embedding of each response generated with MDSK-RAG and the embedding of the corresponding expert reference answer, shown for each evaluation question (k = 5: magenta; k = 10: orange; k = 20: green). We performed the sensitivity analysis of k for the framework using the main model, gemma-2-9b-it.

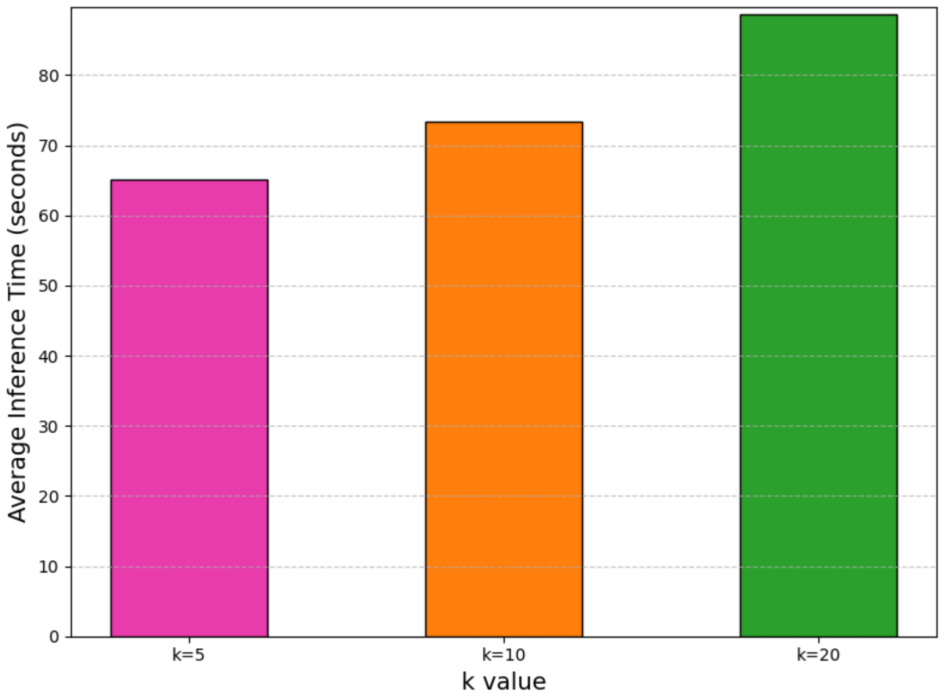

Figure S2 Average inference times with MDSK-RAG (k = 5: magenta; k = 10: orange; k = 20: green). We performed the sensitivity analysis of k for the framework using the main model, gemma-2-9b-it.

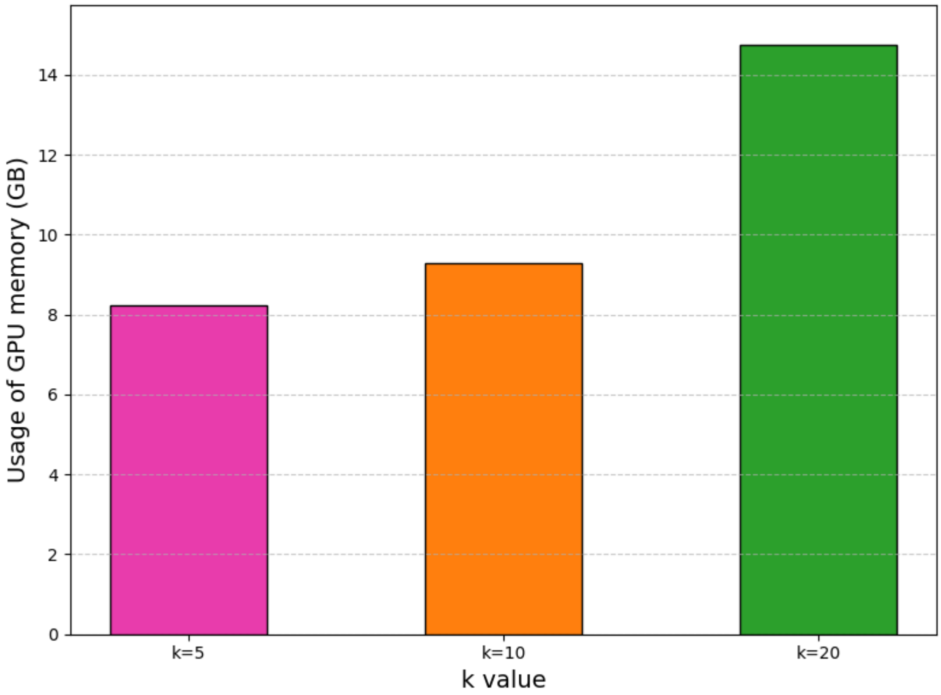

Figure S3 Net GPU memory usage (peak minus baseline idle) required by MDSK-RAG (k = 5: magenta; k = 10: orange; k = 20: green). We performed the sensitivity analysis of k for the framework using the main model, gemma-2-9b-it.

Box S1. Example of answers for the 14 expert-defined questions.

【Question】

1. For metal sulfide photocatalysts containing Ag as a constituent element, please specify three important factors strongly affecting the activity for hydrogen evolution.

【Example of major answer】

① Formation of a solid-solution, and optimization of solid-solution ratios of constituent elements such as Zn^{2+}, Ga^{3+}, In^{3+}, Ge^{4+}, and Sn^{4+} for Ag-based solid solution affect the activity due to change the band structure.

② Loading of cocatalysts such as Rh, Pt, and Ru affects the activity due to function as an active site for hydrogen evolution.

③ Synthesis conditions such as calcination temperature, calcination time, and excess amounts of starting materials affect the activity due to involve with a crystal structure, particle size, particulate morphology and an electronic structure. For a $(AgIn)_{0.22}Zn_{1.56}S_2$ photocatalyst, a heat-treatment condition greatly affects the activity. Heating under vacuum in a sealed quartz ampule tube is effective than that under N_2-flow.

【Example of minor answer】

① Doping of a Ni^{2+} ion into $AgGaS_2$ is effective to improve the activity under visible light with long wavelengths because an impurity level of a Ni^{2+} ion forms in a forbidden band of $AgGaS_2$.

② Particulate morphology affects the activity. A nano-step structure is observed on the surface of a $(AgIn)_{0.22}Zn_{1.56}S_2$ photocatalyst. The specific morphology contributes to efficient charge separation, leading to high activity.

③ Crystal structure affects the activity due to involve with an electronic structure.

【Question】

2. For metal sulfide photocatalysts containing Cu as a constituent element, please specify three important factors strongly affecting the activity for hydrogen evolution.

【Example of major answer】

① Formation of a solid-solution, and optimization of solid-solution ratios of constituent elements such as Zn^{2+}, Ga^{3+}, In^{3+}, Ge^{4+}, Sn^{4+}, V^{5+}, Nb^{5+}, and Ta^{5+} for Cu-based solid solution affect the activity due to change the band structure.

② Loading of cocatalysts such as Rh, Pt, and Ru affects the activity due to function as an active site for hydrogen evolution. For a Cu_2ZnGeS_4 photocatalyst, Ru works an effective cocatalyst rather than Pt and Rh.

③ Synthesis conditions such as calcination temperature, calcination time, and excess amounts of starting materials affect the activity due to involve with a crystal structure, particle size, morphology and an electronic structure.

【Example of minor answer】

① Particulate morphology affects the activity. A specific hexagonal structure where it works as a reduction site is observed on the surface of$(CuIn)_{0.03}Zn_{1.94}S_2$ particles.

② Crystal structure affects the activity due to involve with an electronic structure.

【Question】

3. With respect to AgGaS2, please specify three important factors strongly affecting the activity for hydrogen evolution.

【Example of major answer】

① Doping of a Ni^{2+} ion into $AgGaS_2$ is effective to improve the activity under visible light with long wavelengths because an impurity level of a Ni^{2+} ion forms in a forbidden band of $AgGaS_2$. However, a further increase in a doping amount decreases the activity due to work as recombination centers of photogenerated carriers.

② Loading of cocatalysts such as Rh, Pt, and Ru affects the activity due to function as an active site for hydrogen evolution.

③ Synthesis conditions such as calcination temperature, calcination time, and excess amounts of starting materials affect the activity due to involve with a crystal structure, particle size, morphology and an electronic structure.

【Example of minor answer】

① An activity depends on incident of light wavelengths according to a property of light absorption of the photocatalyst.

② Particulate morphology affects the activity due to contribute to the number of active sites and charge separation.

【Question】

4. For metal sulfide photocatalysts with a chalcopyrite structure, please specify three important factors strongly affecting the activity for hydrogen evolution.

【Example of major answer】

① Formation of a solid-solution, and optimization of solid-solution ratios of constituent elements such as Cu^{+}, Ag^{+}, Zn^{2+}, Ga^{3+}, and In^{3+} affect the activity due to change the band structure.

② Loading of cocatalysts such as Rh, Pt, and Ru affects the activity due to function as an active site for hydrogen evolution.

③ Synthesis conditions such as calcination temperature, calcination time, and excess amounts of starting materials affect the activity due to involve with a crystal structure, particle size, morphology and an electronic structure.

【Example of minor answer】

① Crystal structure affects the activity due to involve with an electronic structure.

② Doping of transition metals such as a Ni^{2+} ion is effective to improve the activity under visible light with long wavelengths. For example, an impurity level of a Ni^{2+} ion forms in a forbidden band of $AgGaS_2$ with a chalcopyrite structure, leading to sensitize the photocatalyst to visible light with long wavelengths.

③ Particulate morphology affects the activity due to contribute to the number of active sites and charge separation.

【Question】

5. For metal sulfide photocatalysts with a stannite structure, please specify three important factors strongly affecting the activity for hydrogen evolution.

【Example of major answer】

① Formation of a solid-solution, and optimization of solid-solution ratios of constituent elements such as Cu^{+}, Ag^{+}, Ge^{4+}, and Sn^{4+} affect the activity due to change the band structure.

② Loading of cocatalysts such as Rh, Pt, and Ru affects the activity due to function as an active site for hydrogen evolution. For a Cu_2ZnGeS_4 photocatalyst with a stannite structure, Ru works an effective cocatalyst rather than Pt and Rh.

③ Synthesis conditions such as calcination temperature, calcination time, and excess amounts of starting materials affect the activity due to involve with a crystal structure, particle size, morphology and an electronic structure.

【Example of minor answer】

① Crystal structure affects the activity due to involve with an electronic structure.

② Particulate morphology affects the activity due to contribute to the number of active sites and charge separation.

【Question】

6. Please enumerate five crystal structures of metal sulfide photocatalysts with a trend of the high activity for hydrogen evolution.

【Example of major answer】

The following crystal structures were selected on the basis of the reported apparent quantum efficiencies.

① Zinc blende structure (e.g. ZnS, CdS, and (ZnS)-(CuCl) solid solution)

② Wurtzite structure (e.g. ZnS, CdS, and (ZnS)-($AgInS_2$) solid solution)

③ Layered structure (e.g. $NaInS_2$, $ZnIn_2S_4$, and $AGa_2In_3S_8$(A=Cu and Ag))

④ Chalcopyrite structure (e.g. $AgGaS_2$, (ZuS)-($CuGaS_2$) and (ZnS)-($CuInS_2$)-($AgInS_2$) solid solutions)

⑤ Stannite structure (e.g. $A(I)_2ZnB(IV)S_4$(A(I)=Cu and Ag; B(IV)=Ge and Sn)

【Example of minor answer】

The following crystal structures were selected on the basis of the reported apparent quantum efficiencies.

⑥ Sulvanite structure (e.g. $Cu_3A(V)S_4$(A(V)=V, Nb, and Ta))

【Question】

7. Please enumerate three cocatalysts of metal sulfide photocatalysts with a trend of the high activity for hydrogen evolution.

【Example of answer】

Pt, Rh, and Ru cocatalysts function as active sites for hydrogen evolution on metal sulfide photocatalysts. In particular, Ru is an effective cocatalyst for hydrogen evolution among those catalysts, which is reported by Tsuji, Kudo and coworkers (Chem. Mater. 2010, 22, 1402.).

【Question】

8. For metal sulfide photocatalysts, please explain the mechanism of which formation of solid-solution affects the activity for hydrogen evolution.

【Example of answer】

① A band structure changes by formation of solid-solution between semiconductor photocatalysts with wide and narrow band gaps, leading to affect a property of light absorption, driving force of reduction of water, and mobilities of photogenerated electrons and holes. Those factors greatly affect the activity.

② Specific particulate morphologies are often observed by formation of solid-solution. For example, a specific hexagonal structure where it works as a reduction site is observed on the surface of $(CuIn)_{0.03}Zn_{1.94}S_2$ particles. In addition, the nano-step structure is observed on the surface of a $(AgIn)_{0.22}Zn_{1.56}S_2$ photocatalyst. The specific morphology contributes to efficient charge separation, leading to high activity.

【Question】

9. Please explain the reason why it is difficult to employ a metal sulfide photocatalyst as a single particle for water splitting.

【Example of answer】

S^{2-} ions containing metal sulfides oxidized by photogenerated holes accompanied with elution of cations consisting of the materials, which is called photocorrosion. Thus, metal sulfides cannot evolve oxygen by oxidation of water. This demerit can be solved by using sacrificial reagents such as S^{2-} or SO_3^{2-} ions. Many metal sulfide photocatalysts evolve hydrogen from an aqueous solution containing these sacrificial reagents under light irradiation. In particular, Cu(I)-containing metal sulfides are promising materials showing a p-type semiconductor character. Although they cannot split water as a single particle, water splitting can be achieved by constructing Z-schematic and photoelectrochemical systems employing n-type semiconductor photocatalysts.

【Question】

10. Please specify several examples of elements constituting the valence band of metal sulfide photocatalysts.

【Example of answer】

① Cu (3d orbitals of Cu^{+} ions)

② Ag (4d orbitals of Ag^{+} ions)

③ S (3p orbitals of S)

④ Mn (3d orbitals of Mn^{2+} ions)

【Question】

11. Please specify several examples of elements constituting the conduction band of metal sulfide photocatalysts.

【Example of answer】

<d^{10} metal ion>

① Zn (4s and 4p orbitals of Zn^{2+} ions)

② Cd (5s and 5p orbitals of Cd^{2+} ions)

③ Ga (4s and 4p orbitals of Ga^{3+} ions)

④ In (5s and 5p orbitals of In^{3+} ions)

⑤ Ge (4s and 4p orbitals of Ge^{4+} ions)

⑥ Sn (5s and 5p orbitals of Sn^{4+} ions)

<d^0 metal ion>

⑦ V (3d orbitals of V^{5+} ions)

⑧ Nb (4d orbitals of Nb^{5+} ions)

⑨ Ta (5d orbitals of Ta^{5+} ions)

【Question】

12. Please specify metal sulfide photocatalysts with response to long wavelength of visible light up to 600 nm and explain the reason why those photocatalysts can response to long wavelength of visible light in terms of their band structures.

【Example of answer】

For example, A_2ZnSnS_4 (A=Cu and Ag) with a stannite structure, $CuGa_2In_3S_8$ and $MnGaInS_4$ with layered structures, $(CuAg)_{0.15}In_{0.3}Zn_{1.4}S_2$ with a chalcopyrite structure, Cu_3VS_3 and $Cu_3Nb_{0.9}V_{0.1}S_4$ with sulvanite structures are the metal sulfide photocatalysts responding to visible light with long wavelengths up to 600 nm. The valence bands of those photocatalysts consist of orbitals of Cu^+, Ag^+, Mn^{2+} and S. The valence bands consisting of Cu^+, Ag^+, and Mn^{2+} ions locate at more negative level than that of S. On the other hand, the conduction bands of the photocatalysts consist of Zn^{2+}, Sn^{4+}, Ga^{3+}, In^{3+}, V^{5+}, and Nb^{5+} ions. In particular, the conduction bands consisting of Sn^{4+}, In^{3+}, and V^{5+} ions locate at more positive levels than those of Zn^{2+}, Ga^{3+}, and Nb^{5+} ions, respectively. Therefore, those photocatalysts possess the narrow band gaps to respond to visible light with long wavelengths.

【Question】

13. For metal sulfide photocatalysts, please specify common features of elements constituting valence band and conduction band, respectively.

【Example of answer】

The valence band of metal sulfide photocatalysts consists of S 3p orbitals. In the case of metal sulfides consisting of such as Cu^{+}, Ag^{+}, and Mn^{2+} ions, the valence bands consisting of those d orbitals locate at more negative position than that of S. For the conduction bands consisting of d^0 metal ions such as V^{5+}, Nb^{5+}, and Ta^{5+}, those d orbitals form the conduction bands. On the other hand, the metal sulfides consisting of d^{10} metal ions such as Zn^{2+}, Cd^{2+}, Ga^{3+}, In^{3+}, Ge^{4+}, and Sn^{4+} form the conduction bands consisting of s and p hybrid orbitals. The detail summary of the valence band and the conduction band is noted as follows.

<Valence band>

① 3d orbitals of Cu^{+} ions

② 4d orbitals of Ag^{+} ions

③ 3p orbitals of S

④ 3d orbitals of Mn^{2+} ions

<Conduction band>

⑤ 4s and 4p orbitals of Zn^{2+} ions

⑥ 5s and 5p orbitals of Cd^{2+} ions

⑦ 4s and 4p orbitals of Ga^{3+} ions

⑧ 5s and 5p orbitals of In^{3+} ions

⑨ 4s and 4p orbitals of Ge^{4+} ions

⑩ 5s and 5p orbitals of Sn^{4+} ions

⑪ 3d orbitals of V^{5+} ions

⑫ 4d orbitals of Nb^{5+} ions

⑬ 5d orbitals of Ta^{5+} ions

【Question】

14. Please specify the reason why development of metal sulfide photocatalysts with respond to long wavelength of visible light is important for practical solar water splitting.

【Example of answer】

Efficient utilization of solar light is a critical issue for practical solar water splitting employing powder-based photocatalysts. Solar-to-hydrogen (STH) efficiency exceeding 10% should be achieved for the practical application. However, it cannot be achieved by only utilizing ultraviolet in solar spectrum because it accounts only 5% of solar energy. Therefore, development of photocatalysts responding to visible light with long wavelengths in solar spectrum is strongly demand. Metal sulfides are promising photocatalytic material groups because many of them (e.g. (ZnS)-($CuInS_2$)-($AgInS_2$), $CuGa_2In_3S_8$, and so on) show relatively high activities for hydrogen evolution under visible light. From the background mentioned above, it is important to develop metal sulfide photocatalysts responding to visible light with long wavelengths for practical application.

Box S2. Comparison of the output responses generated using gemma-2-9b-it with and without MDSK-RAG for the 14 expert-defined questions.

【Question】

1. For metal sulfide photocatalysts containing Ag as a constituent element, please specify three important factors strongly affecting the activity for hydrogen evolution.

【Output responses generated without MDSK-RAG】

Three important factors affecting the activity of Ag-containing metal sulfide photocatalysts for hydrogen evolution:

1. **Composition and Morphology:** The specific type of metal sulfide (e.g., MoS_2, WS_2, CdS) and its morphology (e.g., nanosheets, nanorods, quantum dots) significantly influence the photocatalytic activity. Different metal sulfides possess varying band structures and light absorption properties, while morphology affects the surface area, light scattering, and charge carrier transport.

2. **Ag Content and Distribution:** The amount and distribution of Ag within the metal sulfide matrix play a crucial role. Ag can act as a cocatalyst, facilitating charge separation and reducing the overpotential for hydrogen evolution. However, excessive Ag can lead to aggregation and hinder light absorption.

3. **Surface Modification:** Surface treatments, such as doping with other elements or depositing noble metals, can enhance the photocatalytic performance. Doping can modify the electronic structure and improve charge carrier separation, while noble metal deposition can act as efficient electron sinks, further promoting hydrogen evolution.

【Output responses generated with MDSK-RAG】

Here are three important factors strongly affecting the activity for hydrogen evolution in metal sulfide photocatalysts containing Ag:

1. **Band Gap Energy:** The band gap of the Ag-containing sulfide should be suitable for visible light absorption. A smaller band gap allows for the utilization of a broader range of the solar spectrum, leading to increased light absorption and consequently higher photocatalytic activity.

2. **Charge Carrier Separation Efficiency:** Efficient separation of photogenerated electrons and holes is crucial to prevent recombination and enhance the availability of charge carriers for the hydrogen evolution reaction. Factors influencing this include the crystal structure, morphology, and the presence of co-catalysts.

3. **Surface Area and Morphology:** A larger surface area provides more active sites for the adsorption of reactants and the occurrence of the catalytic reaction. The morphology of the photocatalyst can also influence charge transport and mass

transfer, affecting overall activity.

【Question】

2. For metal sulfide photocatalysts containing Cu as a constituent element, please specify three important factors strongly affecting the activity for hydrogen evolution.

【Output responses generated without MDSK-RAG】

Three important factors affecting the activity of Cu-based metal sulfide photocatalysts for hydrogen evolution:

1. **Band Structure and Energy Levels:** The position of the valence band (VB) and conduction band (CB) edges relative to the redox potentials of water splitting reactions (H+/H2 and O2/H2O) is crucial. A suitable band gap and aligned energy levels ensure efficient charge separation and transfer, promoting the reduction of protons to hydrogen.

2. **Morphology and Surface Area:** A high surface area and well-defined morphology (e.g., nanowires, nanosheets) provide more active sites for light absorption and catalytic reactions. This enhances the interaction between the photocatalyst and reactants, accelerating the hydrogen evolution process.

3. **Composition and Doping:** The specific metal sulfide composition (e.g., CuS, CuInS2) and the presence of dopants (e.g., S, N, O) can significantly influence the electronic properties, light absorption, and catalytic activity. Doping can modify the band structure, introduce defect states, and enhance charge carrier separation, ultimately boosting hydrogen evolution efficiency.

【Output responses generated with MDSK-RAG】

For metal sulfide photocatalysts containing Cu as a constituent element, three important factors strongly affecting the activity for hydrogen evolution are:

1. **Crystal Structure:** The specific crystal structure of the copper sulfide material significantly influences its electronic properties and thus its photocatalytic activity. For example, the sulvanite structure (found in Cu3VS4, Cu3NbS4, and Cu3TaS4) has been shown to be more favorable for hydrogen evolution compared to other structures like covellite (found in CuS).

2. **Composition and Doping:** The precise composition of the copper sulfide, including the presence of other elements like vanadium, niobium, tantalum, tin, or germanium, can dramatically affect its band gap, charge carrier separation efficiency, and overall photocatalytic performance. Doping with specific elements can further tune these properties.

3. **Cocatalysts:** The addition of cocatalysts, such as ruthenium (Ru), can significantly enhance the hydrogen evolution activity of copper sulfide photocatalysts. These cocatalysts can act as active sites for the reduction of protons to hydrogen gas,

facilitating the overall reaction.

Let me know if you have any other questions.

【Question】

3. With respect to AgGaS2, please specify three important factors strongly affecting the activity for hydrogen evolution.

【Output responses generated without MDSK-RAG】

AgGaS2 is a promising material for hydrogen evolution reaction (HER) due to its unique properties. However, its activity can be significantly influenced by several factors. Here are three important ones:

1. **Surface Morphology and Structure:** The surface area, roughness, and crystallographic facets of AgGaS2 directly impact its catalytic activity. A higher surface area provides more active sites for the HER, while specific crystal facets can exhibit enhanced catalytic performance due to their unique electronic and geometric properties.

2. **Doping and Compositional Engineering:** Introducing dopants or modifying the AgGaS2 composition can significantly alter its electronic structure and catalytic properties. For example, doping with transition metals can create defect sites that act as active centers for HER, while adjusting the Ag:Ga ratio can influence the band gap and charge carrier concentration.

3. **Electrolyte Conditions:** The choice of electrolyte, pH, and applied potential can significantly affect the HER activity of AgGaS2. The electrolyte's conductivity, acidity/basicity, and the presence of specific ions can influence the reaction kinetics and mass transport, ultimately impacting the overall catalytic performance.

Understanding and optimizing these factors is crucial for maximizing the HER activity of AgGaS2 and its potential for practical applications in hydrogen production.

【Output responses generated with MDSK-RAG】

Based on the provided information, here are three important factors strongly affecting the activity for hydrogen evolution in AgGaS2:

1. **Cocatalysts:** The presence and concentration of cocatalysts, particularly Ru-based ones, significantly enhance the hydrogen evolution rate. This suggests that cocatalysts play a crucial role in facilitating the adsorption and activation of reactants (e.g., water molecules) and/or in accelerating the rate-limiting steps of the hydrogen evolution reaction.

2. **Doping:** The text highlights that doping AgGaS2 with nickel improves its photocatalytic performance. This likely occurs due to the creation of an electron donor level within the material's band structure, enabling better visible light absorption and facilitating the reduction of water molecules.

3. **Light Irradiation:** The photocatalytic activity of AgGaS2 is directly dependent on light irradiation. The text mentions that visible light (λ> 420 nm) is used for irradiation, and the rate of hydrogen evolution is influenced by the intensity and wavelength of the light source.

【Question】

4. For metal sulfide photocatalysts with a chalcopyrite structure, please specify three important factors strongly affecting the activity for hydrogen evolution.

【Output responses generated without MDSK-RAG】

Three important factors strongly affecting the activity for hydrogen evolution in metal sulfide photocatalysts with a chalcopyrite structure:

1. **Band Gap Energy:** The band gap energy of the photocatalyst determines the range of light it can absorb. A smaller band gap allows absorption of a wider range of light, including visible light, which is more abundant and readily available. This leads to increased photogenerated electron-hole pairs, enhancing hydrogen evolution.

2. **Surface Morphology and Area:** A high surface area provides more active sites for the adsorption of reactants (e.g., water molecules) and the subsequent reaction steps involved in hydrogen evolution. Nanostructured morphologies like nanowires, nanotubes, or nanosheets can significantly increase the surface area compared to bulk materials.

3. **Charge Carrier Separation and Transport:** Efficient separation and transport of photogenerated electrons and holes are crucial for preventing recombination and maximizing the number of charge carriers available for the hydrogen evolution reaction. Doping, heterojunction formation, or surface modification can be employed to improve charge carrier separation and transport.

Let me know if you'd like to explore any of these factors in more detail!

【Output responses generated with MDSK-RAG】

Here are three important factors strongly affecting the activity for hydrogen evolution in metal sulfide photocatalysts with a chalcopyrite structure:

1. **Composition:** The specific combination of metal and sulfur elements within the chalcopyrite structure significantly influences the band gap, charge carrier mobility, and overall photocatalytic efficiency. Different ternary combinations can lead to variations in light absorption, charge separation, and reaction kinetics, ultimately affecting hydrogen evolution rates.

2. **Defect Engineering:** Intentionally introducing defects into the chalcopyrite lattice can act as active sites for charge carrier trapping and recombination, enhancing the separation of photogenerated electrons and holes. This improved charge separation can lead to a higher number of available charge carriers for participating in the hydrogen evolution reaction.

3. **Surface Morphology and Area:** The surface properties of the photocatalyst, including its morphology (e.g., nanorods, nanosheets) and surface area, play a crucial role in determining the number of active sites available for the reaction. A larger surface area and a more favorable morphology can provide more sites for the adsorption of reactants and the subsequent catalytic process, leading to enhanced hydrogen evolution activity.

【Question】

5. For metal sulfide photocatalysts with a stannite structure, please specify three important factors strongly affecting the activity for hydrogen evolution.

【Output responses generated without MDSK-RAG】

Three important factors strongly affecting the activity for hydrogen evolution in stannite metal sulfide photocatalysts:

1. **Band Gap Energy:** The band gap energy should be suitable for absorbing visible light. A smaller band gap allows for broader light absorption, leading to increased photogenerated electron-hole pairs and ultimately higher hydrogen evolution rates.

2. **Charge Carrier Separation Efficiency:** Efficient separation of photogenerated electrons and holes is crucial to prevent recombination and maximize the number of charge carriers available for hydrogen evolution. This can be influenced by factors like the composition of the stannite structure, the presence of heterojunctions, and surface modifications.

3. **Surface Area and Morphology:** A larger surface area provides more active sites for the adsorption of reactants and the catalytic reaction. Additionally, specific morphologies, such as nanowires or nanosheets, can enhance charge transport and expose more active sites, further boosting the photocatalytic activity.

【Output responses generated with MDSK-RAG】

Based on the provided information, here are three important factors strongly affecting the activity for hydrogen evolution in metal sulfide photocatalysts with a stannite structure:

1. **Composition:** The specific elements and their ratios within the stannite structure significantly influence the electronic properties, band gap, and light absorption characteristics of the photocatalyst. For example, the paper highlights the impact of varying AII (Cu and Ag) and AIV (Sn and Ge) elements on the photocatalytic activity.

2. **Band Gap:** The band gap of the photocatalyst determines the range of light wavelengths it can absorb. A smaller band gap allows for absorption of visible light, which is crucial for solar-driven hydrogen evolution.

3. **Surface Area and Morphology:** A larger surface area provides more active sites for the adsorption of reactants and the occurrence of photocatalytic reactions. The morphology of the photocatalyst (e.g., nanostructures, porous materials) can also enhance surface area and facilitate mass transport.

Let me know if you have any other questions.

【Question】

6. Please enumerate five crystal structures of metal sulfide photocatalysts with a trend of the high activity for hydrogen evolution.

【Output responses generated without MDSK-RAG】

It is difficult to definitively rank metal sulfide photocatalysts by activity for hydrogen evolution without extensive experimental data.

However, I can provide five common crystal structures of metal sulfide photocatalysts known for their potential in hydrogen evolution, along with factors that generally contribute to higher activity:

1. ZnS (Wurtzite)

* **Structure:** Hexagonal close-packed with zinc and sulfur atoms arranged in a specific pattern.
* **Activity Trends:**
    * Doping with other elements (e.g., Cu, Ag) can enhance charge separation and reduce recombination.
    * Nanostructuring (nanoparticles, nanowires) increases surface area and light absorption.

2. CdS (Cubic)

* **Structure:** Face-centered cubic lattice.
* **Activity Trends:**

* Quantum dots (nanocrystals) of CdS exhibit size-dependent activity, with smaller sizes generally leading to higher activity.

* Surface modification with organic molecules can improve charge transfer and stability.

3. MoS₂ (Layered)

* **Structure:** Two-dimensional sheets of molybdenum and sulfur atoms arranged in a hexagonal lattice.
* **Activity Trends:**
    * Edge sites on MoS₂ are more active for hydrogen evolution than basal plane sites.
    * Defects and vacancies within the MoS₂ layers can enhance catalytic activity.

4. Cu₂S (Chalcopyrite)

* **Structure:** Tetragonal crystal structure with copper and sulfur atoms arranged in a specific pattern.
* **Activity Trends:**
    * Cu₂S nanostructures with high surface area and exposed active sites show promising activity.
    * Doping with other metals (e.g., Ag, Au) can improve charge transport and stability.

5. NiS (Hexagonal)

* **Structure:** Close-packed hexagonal lattice.
* **Activity Trends:**
    * NiS nanostructures with controlled morphology and composition exhibit enhanced activity.
    * Doping with other elements (e.g., Co, Fe) can modify electronic properties and catalytic performance.

General Trends for Higher Activity:

* **High Surface Area:** Nanostructures (nanoparticles, nanowires, nanotubes) provide more active sites for the reaction.
* **Efficient Charge Separation:** Doping, heterojunctions, or surface modifications can reduce electron-hole recombination

【Output responses generated with MDSK-RAG】

It is difficult to definitively rank the five most active metal sulfide photocatalysts for hydrogen evolution based solely on the provided information. The text highlights several materials with promising activity, but a direct comparison of their performance is lacking.

However, based on the text's descriptions and the general trends in photocatalysis, here are five potential candidates with a

trend towards higher activity:

1. **(AgIn)0.22Zn1.56S2:** This chalcopyrite-type material exhibited the highest hydrogen evolution rate (748 μmol h-1) among the tested materials.
2. **Stannite-type complex sulfides (A₂-Zn-AIV-S₄):** The research specifically highlights the high activity of these novel materials, attributing it to their unique surface nanostep structure and the absence of photocorrosive CdS modifiers.
3. **Zn2GeS4:** This material showed improved activity with the addition of a Ru(0.5%) cocatalyst.
4. **La2S3:** While its specific activity is not mentioned, La2S3 is a well-known photocatalyst for hydrogen evolution.
5. **Covellite (CuS):** Although CuS did not show activity in the specific study mentioned, it is a common metal sulfide photocatalyst that has been shown to be active for hydrogen evolution under certain conditions.

Trend:

The trend towards higher activity generally involves:

* **Defect engineering:** Introducing defects into the crystal structure can enhance light absorption and charge separation.
* **Surface modification:** Modifying the surface with cocatalysts or other functional groups can improve charge transfer and reduce recombination.
* **Unique crystal structures:** Materials with specific surface features or electronic properties, such as the stannite-type complex sulfides, can exhibit enhanced activity.

It's important to note that this is a general trend, and the specific performance of a material depends on various factors, including synthesis conditions, reaction parameters, and the specific application.

【Question】

7. Please enumerate three cocatalysts of metal sulfide photocatalysts with a trend of the high activity for hydrogen evolution.

【Output responses generated without MDSK-RAG】

It's difficult to definitively rank cocatalysts in terms of "highest activity" for hydrogen evolution because performance depends on various factors like the specific metal sulfide, reaction conditions, and characterization methods.

However, here are three cocatalysts commonly used with metal sulfide photocatalysts for hydrogen evolution, along with general trends in their activity:

1. **Pt:** Platinum is a well-established cocatalyst for hydrogen evolution due to its excellent catalytic activity and stability. It often serves as a benchmark for comparison with other cocatalysts.

2. **MoS₂:** Molybdenum disulfide (MoS₂) is a promising cocatalyst that exhibits good catalytic activity and can be synthesized via various methods, making it more cost-effective than Pt.

3. **NiFe-LDH:** Nickel-iron layered double hydroxides (NiFe-LDH) are emerging as efficient cocatalysts due to their synergistic effect, good conductivity, and ability to promote charge separation.

Trend in Activity:

While Pt generally shows high activity, research is actively exploring alternative cocatalysts like MoS₂ and NiFe-LDH to improve cost-effectiveness and potentially surpass Pt's performance. The specific activity of each cocatalyst depends on factors like its morphology, loading, and interaction with the metal sulfide photocatalyst.

It's important to note that this is a simplified overview, and the field of photocatalysis is constantly evolving with new materials and strategies being developed.

【Output responses generated with MDSK-RAG】

Based on the provided information, here are three cocatalysts of metal sulfide photocatalysts with a trend of high activity for hydrogen evolution:

1. **Pt:** Used in GaS photocatalyst, achieving 252-633 μmol h-1 of hydrogen evolution.
2. **Ru:** Employed in Cu3NbS4 photocatalyst, leading to 273-564 μmol h-1 of hydrogen evolution.
3. **Ru:** Also used in Cu0.54Ag0.36Ga0.54In0.36Zn0.2S2 photocatalyst, resulting in the highest activity of 2358 μmol h-1.

Trend:

The trend suggests that **Ru** cocatalyst exhibits higher activity compared to **Pt** for hydrogen evolution in sulfide photocatalysts.

It's important to note that this trend is based on a limited number of examples and further research is needed to confirm this general trend.

【Question】

8. For metal sulfide photocatalysts, please explain the mechanism of which formation of solid-solution affects the activity for hydrogen evolution.

【Output responses generated without MDSK-RAG】

Mechanism of Solid-Solution Formation and its Effect on Hydrogen Evolution

Metal sulfide photocatalysts have gained significant attention for their potential in solar-driven hydrogen evolution. The formation of solid solutions within these materials can significantly influence their photocatalytic activity.

1. Mechanism of Solid-Solution Formation:

Solid solutions are formed when two or more different metal sulfides are chemically combined in a single crystal lattice. This can occur through various methods, including:

* **Co-precipitation:** Simultaneous precipitation of different metal sulfides from a solution.
* **Hydrothermal synthesis:** Reaction of metal precursors in a high-temperature, high-pressure aqueous environment.
* **Solid-state reaction:** Direct reaction of metal sulfide powders at elevated temperatures.

The specific method used depends on the desired composition and properties of the solid solution.

2. Effect on Hydrogen Evolution:

The formation of solid solutions can enhance hydrogen evolution through several mechanisms:

* **Bandgap Engineering:** By combining different metal sulfides, the bandgap of the resulting solid solution can be tuned to match the solar spectrum more effectively. This allows for better absorption of sunlight and increased photocatalytic activity.
* **Charge Carrier Separation:** Solid solutions can promote charge carrier separation by creating heterojunctions between different metal sulfide phases. This reduces electron-hole recombination and enhances the efficiency of charge transfer to the reaction sites.
* **Surface Area Enhancement:** The formation of solid solutions can lead to an increase in the surface area of the photocatalyst, providing more active sites for hydrogen evolution reactions.
* **Synergistic Effects:** Different metal sulfides can exhibit synergistic effects when combined in a solid solution. For example, one metal sulfide may act as a good electron donor, while another acts as a good hole acceptor, leading to enhanced charge transfer and catalytic activity.

3. Examples:

Several studies have demonstrated the positive impact of solid-solution formation on hydrogen evolution using metal sulfide photocatalysts. For example, solid solutions of CdS-ZnS and MoS2-WS2 have shown improved photocatalytic activity compared to their individual components.

Conclusion:

The formation of solid solutions within metal sulfide photocatalysts offers a promising strategy for enhancing their hydrogen evolution performance. By carefully controlling the composition and structure of the solid solution, researchers can optimize the bandgap, charge carrier separation, surface area, and synergistic effects to achieve high photocatalytic efficiency.

【Output responses generated with MDSK-RAG】

The formation of a solid solution of ZnS–CuInS2–AgInS2 likely enhances the activity for hydrogen evolution through several mechanisms:

1. **Band Gap Engineering:** The incorporation of CuInS2 and AgInS2 into the ZnS lattice can modify the band gap of the resulting solid solution. This can lead to better absorption of visible light, which is crucial for driving the photocatalytic reaction.

2. **Improved Charge Separation:** The presence of different semiconductor components in the solid solution can create heterojunctions. These heterojunctions can facilitate the separation of photogenerated electron-hole pairs, reducing recombination and enhancing the efficiency of charge transfer to the surface for hydrogen evolution.

3. **Enhanced Surface Area:** The formation of a solid solution can lead to the creation of a more porous and nanostructured surface. This increased surface area provides more active sites for the adsorption of reactants and the catalytic reaction.

4. **Synergistic Effects:** The different components in the solid solution may exhibit synergistic effects, where the combined properties are greater than the sum of their individual parts. This can further enhance the overall photocatalytic activity.

Specific to the ZnS–CuInS2–AgInS2 system:

* **CuInS2 and AgInS2:** These materials are known to have good visible-light absorption properties and can act as efficient electron donors.
* **ZnS:** ZnS can act as a good electron acceptor and can also provide structural support for the solid solution.

The combination of these materials in a solid solution likely creates a highly efficient system for visible-light-driven hydrogen

evolution.

It's important to note that the specific details of the synthesis method and the resulting crystal structure and morphology of the solid solution will also play a significant role in determining its photocatalytic activity.

【Question】

9. Please explain the reason why it is difficult to employ a metal sulfide photocatalyst as a single particle for water splitting.

【Output responses generated without MDSK-RAG】

Metal sulfide photocatalysts are promising materials for water splitting due to their visible light absorption and tunable electronic properties. However, they often face challenges when used as single particles. Here's why:

1. Rapid Charge Recombination:

* **High Surface Area:** Single particles have a very high surface area to volume ratio. This exposes more active sites for charge carrier (electrons and holes) generation, but it also increases the probability of these carriers recombining before they can participate in redox reactions (splitting water).
* **Surface Defects:** Single particles often have more surface defects compared to larger structures. These defects act as recombination centers, further accelerating the loss of photogenerated charge carriers.

2. Limited Stability:

* **Corrosion:** Many metal sulfides are susceptible to corrosion in aqueous environments, especially under the acidic or basic conditions required for water splitting. This degradation can lead to a loss of catalytic activity and structural integrity.
* **Photocorrosion:** Photogenerated holes can oxidize the sulfide ions in the catalyst, leading to its degradation and loss of activity.

3. Low Quantum Efficiency:

* **Inefficient Light Absorption:** While metal sulfides can absorb visible light, their overall light absorption efficiency might be limited, resulting in a low number of charge carriers generated per incident photon.
* **Limited Charge Transfer:** Even if charge carriers are generated, their transfer to the surface active sites for water splitting reactions might be inefficient.

4. Difficulty in Separation and Recovery:

* **Small Size:** The small size of single particles makes them difficult to separate from the reaction mixture after water splitting. This can hinder the reusability of the catalyst.

Overcoming these challenges:

Researchers are exploring various strategies to overcome these limitations, such as:

* **Doping:** Introducing other elements into the metal sulfide lattice can modify its electronic properties and reduce charge recombination.
* **Surface Modification:** Coating the metal sulfide with protective layers or modifying its surface with functional groups can enhance its stability and charge transfer efficiency.
* **Composite Materials:** Combining metal sulfides with other materials, such as semiconductors or metal oxides, can create synergistic effects that improve their overall performance.
* **Nanostructuring:** Designing metal sulfide nanostructures with specific morphologies (e.g., nanowires, nanotubes) can enhance their light absorption, charge separation, and catalytic activity.

By addressing these challenges, metal sulfide photocatalysts hold great potential for efficient and sustainable water splitting.

【Output responses generated with MDSK-RAG】
The text you provided highlights several reasons why employing a metal sulfide photocatalyst as a single particle for water splitting is difficult:

* **Photocorrosion:** Metal sulfide photocatalysts are susceptible to photocorrosion, where they degrade under light irradiation due to oxidation reactions. This reduces their efficiency and lifespan.
* **Limited Visible Light Absorption:** Many metal sulfides have a band gap that primarily absorbs ultraviolet light, limiting their efficiency in utilizing the more abundant visible light for water splitting.
* **Low Charge Separation Efficiency:** Metal sulfide photocatalysts often struggle to efficiently separate the photogenerated electrons and holes, leading to recombination and reduced photocatalytic activity.

Strategies to Overcome These Challenges:

The text proposes two main strategies to address these challenges:

1. **Z-scheme System:** Combining a H2-evolving metal sulfide photocatalyst with an O2-evolving photocatalyst and an

electron mediator can enhance charge separation and improve overall efficiency.

2. **Photoelectrochemical Cell:** Utilizing metal sulfides as p-type photocathodes in a photoelectrochemical cell can facilitate water reduction to hydrogen in a more controlled and efficient manner.

Let me know if you have any other questions.

【Question】

10. Please specify several examples of elements constituting the valence band of metal sulfide photocatalysts.

【Output responses generated without MDSK-RAG】

Metal sulfide photocatalysts are promising materials for solar energy conversion due to their tunable band gaps and excellent stability. The valence band (VB) plays a crucial role in the photocatalytic process, as it determines the oxidation potential and the type of redox reactions that can occur.

To provide specific examples, please consider the following:

* **Metal:** Different metals (e.g., Cu, Zn, Cd, Mo) can significantly influence the VB composition.
* **Sulfur species:** Various sulfur species (e.g., S^{2-}, S, S^0) can contribute to the VB structure.
* **Crystal structure:** The arrangement of metal and sulfur atoms in the crystal lattice affects the VB energy levels.

By considering these factors, you can identify specific elements that constitute the VB of different metal sulfide photocatalysts.

* **Cu_2S:** The VB of Cu_2S is primarily composed of **Cu 3d orbitals** and **S 3p orbitals**. The hybridization of these orbitals creates a band structure with a relatively high VB edge, suitable for oxidizing water molecules.

* **ZnS:** In ZnS, the VB is mainly formed by **Zn 3d orbitals** and **S 3p orbitals**. The strong ionic character of the Zn-S bond contributes to a wider band gap and a more positive VB edge compared to Cu_2S.

* **CdS:** Similar to ZnS, the VB of CdS is dominated by **Cd 4d orbitals** and **S 3p orbitals**. However, the larger size of Cd compared to Zn results in a slightly lower VB edge.

* **MoS_2:** This layered material exhibits a unique VB structure due to the presence of **Mo 4d orbitals** and **S 3p orbitals**. The strong covalent bonding between Mo and S atoms leads to a narrow band gap and a more negative VB edge

compared to other metal sulfides.

These examples demonstrate the diversity of elements contributing to the VB of metal sulfide photocatalysts and how their specific arrangement and hybridization influence the material's photocatalytic properties.

【Output responses generated with MDSK-RAG】

Based on the provided information, here are some examples of elements constituting the valence band of metal sulfide photocatalysts:

* **Cu (Copper):** Frequently found in copper-based sulfides like Cu3NbS4, (AgIn)0.22Zn1.56S2, (AgGa)0.3Zn1.4S2, (CuIn)0.09Zn1.82S2, and CuS.
* **Ag (Silver):** Present in some chalcopyrite-structured sulfides like (AgIn)0.22Zn1.56S2, (AgGa)0.3Zn1.4S2.
* **In (Indium):** Found in chalcopyrite-structured sulfides like (AgIn)0.22Zn1.56S2, (AgGa)0.3Zn1.4S2.
* **Ga (Gallium):** Present in chalcopyrite-structured sulfides like (AgGa)0.3Zn1.4S2.
* **Zn (Zinc):** A common component in chalcopyrite-structured sulfides like (AgIn)0.22Zn1.56S2, (AgGa)0.3Zn1.4S2, (CuIn)0.09Zn1.82S2.
* **Nb (Niobium):** Found in the sulvanite-structured Cu3NbS4.
* **Bi (Bismuth):** Mentioned as a potential element contributing to the valence band in metal sulfide photocatalysts.
* **Pb (Lead):** Mentioned as a potential element contributing to the valence band in metal sulfide photocatalysts.
* **Sn (Tin):** Mentioned as a potential element contributing to the valence band in metal sulfide photocatalysts.
* **S (Sulfur):** The essential component forming the sulfide bonds in all these materials.

The specific combination and arrangement of these elements within the crystal lattice determine the electronic structure and ultimately the photocatalytic properties of the metal sulfide material.

【Question】

11. Please specify several examples of elements constituting the conduction band of metal sulfide photocatalysts.

【Output responses generated without MDSK-RAG】

Metal sulfide photocatalysts are promising materials for solar energy conversion and environmental remediation. Their conduction band (CB) plays a crucial role in electron transfer processes, influencing their photocatalytic efficiency.

To provide specific examples, I need to know which metal sulfide photocatalysts you are interested in.

For instance, are you interested in:

* **Common metal sulfides like ZnS, CdS, or MoS2?**

* **Rare earth metal sulfides like EuS or Gd2S3?**
* **Layered metal sulfides like WS2 or FeS2?**

Once you specify the type of metal sulfide photocatalysts, I can provide examples of elements constituting their conduction bands.

【Output responses generated with MDSK-RAG】
The conduction band of metal sulfide photocatalysts is primarily composed of **d-orbitals** from the metal cations.

Here are some specific examples of elements that contribute to the conduction band:

* **Cu (Copper):** Often present as Cu(I) in these photocatalysts, contributing d10 electrons to the conduction band.
* **Ag (Silver):** Similar to Cu, Ag(I) contributes d10 electrons to the conduction band.
* **Zn (Zinc):** Typically found as Zn(II) with a d10 configuration, contributing to the conduction band.
* **Sn (Tin):** Can exist as Sn(II) or Sn(IV) in these materials, with Sn(II) contributing d10 electrons to the conduction band.
* **Ga (Gallium):** Often incorporated as Ga(III) with a d10 configuration, contributing to the conduction band.
* **In (Indium):** Similar to Ga, In(III) contributes d10 electrons to the conduction band.

The specific composition and arrangement of these metal cations within the crystal lattice determine the energy levels and properties of the conduction band, ultimately influencing the photocatalytic activity of the material.

【Question】
12. Please specify metal sulfide photocatalysts with response to long wavelength of visible light up to 600 nm and explain the reason why those photocatalysts can response to long wavelength of visible light in terms of their band structures.

【Output responses generated without MDSK-RAG】
Here are some metal sulfide photocatalysts that exhibit good visible light absorption and their band structure explanations:

1. Cadmium Sulfide (CdS):

* **Band Gap:** ~2.4 eV
* **Absorption Edge:** ~517 nm (green light)
* **Explanation:** CdS has a direct band gap, meaning electrons can directly transition from the valence band to the conduction band upon light absorption. Its band gap of 2.4 eV allows it to absorb visible light up to around 517 nm.

2. Zinc Sulfide (ZnS):

* **Band Gap:** ~3.37 eV
* **Absorption Edge:** ~365 nm (UV light)
* **Explanation:** ZnS has a wide band gap, making it primarily responsive to UV light. However, by doping or forming quantum dots, its absorption can be extended into the visible region.

3. Copper Indium Sulfide (CuInS2):

* **Band Gap:** ~1.5 eV
* **Absorption Edge:** ~820 nm (near-infrared light)
* **Explanation:** CuInS2 has a narrow band gap, allowing it to absorb a wide range of visible light, including near-infrared wavelengths.

4. Molybdenum Disulfide (MoS2):

* **Band Gap:** ~1.8 eV
* **Absorption Edge:** ~690 nm (red light)
* **Explanation:** MoS2 is a layered material with a direct band gap. Its band structure allows for efficient light absorption in the visible region, particularly in the red light range.

5. Tungsten Disulfide (WS2):

* **Band Gap:** ~2.1 eV
* **Absorption Edge:** ~590 nm (green light)
* **Explanation:** WS2 is another layered material with a direct band gap. Its band structure enables it to absorb visible light up to around 590 nm.

Note: The specific absorption properties of these materials can be further tuned by factors such as particle size, morphology, and surface modifications.

Key Factors for Long Wavelength Visible Light Response:

* **Narrow Band Gap:** Materials with smaller band gaps can absorb lower energy photons, which correspond to longer wavelengths of light.
* **Direct Band Gap:** Direct band gap materials allow for efficient electron-hole pair generation upon light absorption.
* **Quantum Con

【Output responses generated with MDSK-RAG】

Based on the provided information, it's difficult to pinpoint specific metal sulfide photocatalysts that respond to long wavelengths up to 600 nm with certainty. The texts focus on general trends and compositions rather than specific examples with detailed optical characterization data.

However, we can discuss the **general principles** that enable metal sulfide photocatalysts to respond to longer wavelengths of visible light:

* **Band Gap Engineering:** The key factor is the **band gap** of the material. A smaller band gap allows the absorption of lower energy photons, which correspond to longer wavelengths.

    * **Chalcopyrite and Wurtzite Structures:** These structures are often found in metal sulfide photocatalysts and can be tuned by varying the composition. For example, incorporating elements like Ag, In, Cu, or Zn can modify the band gap.
    * **Sulvanite Structures:** These layered structures can also exhibit tunable band gaps.

* **Defect Engineering:** Introducing defects into the crystal lattice can create localized energy states within the band gap, facilitating absorption of photons with energies slightly lower than the band gap.

* **Surface Modification:** Coating the photocatalyst with other materials or modifying the surface chemistry can enhance light absorption and charge separation.

To find specific examples of metal sulfide photocatalysts with a response to 600 nm light, you would need to look for research papers that:

1. **Specifically report the optical absorption spectra** of the materials under investigation.
2. **Characterize the band gap** of the photocatalysts using techniques like UV-Vis spectroscopy or photoluminescence.
3. **Discuss the relationship between the band gap and the wavelength of light absorbed.**

Let me know if you have any other questions.

【Question】

13. For metal sulfide photocatalysts, please specify common features of elements constituting valence band and conduction band, respectively.

【Output responses generated without MDSK-RAG】

Valence Band (VB)

* **Common Elements:** Typically composed of **oxygen (O)** and **sulfur (S)**.
* **Electronic Configuration:** The VB is primarily filled with electrons from the **p orbitals** of these elements.

Conduction Band (CB)

* **Common Elements:** The CB is primarily composed of **metal d orbitals**.
* **Electronic Configuration:** The CB is filled with electrons from the **d orbitals** of the metal cation.

Key Points:

* The energy difference between the VB and CB determines the **band gap** of the photocatalyst.
* The **position of the VB and CB edges** relative to the redox potentials of water and other reactants influences the photocatalytic activity.
* The **nature of the metal cation** significantly affects the electronic properties and photocatalytic performance.

Let me know if you have any other questions.

【Output responses generated with MDSK-RAG】
For metal sulfide photocatalysts, the valence band is typically composed of **sulfur (S) 3p orbitals**, while the conduction band is primarily formed by **metal d orbitals**.

Valence Band:

* **Sulfur (S) 3p orbitals:** These orbitals are responsible for the highest occupied molecular orbital (HOMO) in the valence band. The energy level of the S 3p orbitals is influenced by the electronegativity of the metal ions and the bonding environment.

Conduction Band:

* **Metal d orbitals:** These orbitals are responsible for the lowest unoccupied molecular orbital (LUMO) in the conduction band. The energy level of the metal d orbitals is influenced by the electronic configuration of the metal ion, the crystal field splitting, and the bonding with sulfur.

The specific energy levels of the valence and conduction bands determine the band gap of the photocatalyst, which influences its absorption spectrum and photocatalytic activity.

【Question】

14. Please specify the reason why development of metal sulfide photocatalysts with respond to long wavelength of visible light is important for practical solar water splitting.

【Output responses generated without MDSK-RAG】

Here's why developing metal sulfide photocatalysts that respond to long-wavelength visible light is crucial for practical solar water splitting:

* **Maximizing Solar Energy Utilization:** The solar spectrum contains a significant portion of long-wavelength visible light. Traditional photocatalysts often only absorb a narrow range of UV-Vis light, wasting a large portion of the available solar energy. By extending the absorption range to longer wavelengths, metal sulfide photocatalysts can harness a broader spectrum of sunlight, leading to higher efficiency in water splitting.

* **Reducing Material Costs:** Many efficient photocatalysts, like titanium dioxide (TiO2), require expensive doping or complex synthesis methods to achieve visible light absorption. Metal sulfides, on the other hand, are relatively abundant and inexpensive materials. Developing visible light-active metal sulfides could significantly reduce the cost of solar water splitting technology.

* **Environmental Benefits:** Solar water splitting offers a clean and sustainable way to produce hydrogen fuel, which can replace fossil fuels and reduce greenhouse gas emissions. Making this technology more efficient and affordable through the use of metal sulfide photocatalysts would accelerate the transition to a greener energy future.

Specific Challenges:

While metal sulfides hold great promise, there are challenges to overcome:

* **Stability:** Some metal sulfides are susceptible to photocorrosion (degradation) under prolonged exposure to sunlight and water. Research is focused on developing more stable materials and protective coatings.
* **Charge Separation:** Efficiently separating the photogenerated electrons and holes is crucial for driving the water splitting reactions. Strategies like heterojunctions and surface modifications are being explored to enhance charge separation.

Let me know if you have any other questions!

【Output responses generated with MDSK-RAG】

The development of metal sulfide photocatalysts that respond to long wavelengths of visible light is crucial for practical solar

water splitting for several reasons:

* **Enhanced Light Absorption:** The solar spectrum is rich in visible light, particularly in the longer wavelengths. Metal sulfide photocatalysts with extended absorption into the visible region can harness a larger portion of the solar energy, leading to higher efficiency in water splitting.

* **Reduced Cost:** Traditional semiconductor photocatalysts, like TiO2, require UV light for activation, necessitating expensive UV lamps for practical applications. Utilizing visible light, which is abundant and readily available, significantly reduces the cost and energy requirements of the process.

* **Environmental Friendliness:** Solar water splitting using visible-light-active photocatalysts offers a clean and sustainable approach to hydrogen production. It avoids the use of fossil fuels and reduces greenhouse gas emissions.

In summary, developing metal sulfide photocatalysts that efficiently utilize long-wavelength visible light is essential for making solar water splitting a viable and cost-effective technology for clean hydrogen production.

Box S3. Related information retrieved and summarized from the CSV and PDF data via MDSK-RAG using gemma-2-9b-it for the 14 expert-defined questions.

【Question】

1. For metal sulfide photocatalysts containing Ag as a constituent element, please specify three important factors strongly affecting the activity for hydrogen evolution.

【Related Information】

From experimental data:

This text describes the synthesis, crystal structure, and hydrogen evolution activity of various ternary chalcopyrite and stannite sulfide photocatalysts.

Synthesis Methods:

Most materials were synthesized via solid-state reactions, involving mixing starting materials, sealing them in a quartz ampule, and heat-treating under vacuum. Some precursors were prepared by precipitation from aqueous solutions before heat-treatment.

Crystal Structures:

The dominant crystal structure observed was the **chalcopyrite structure**, found in AgLaOS, Ag0.22In0.22Zn1.56S2, AgGaS2, (AgGa)0.3Zn1.4S2, AgInS2, (AgIn)0.22Zn1.56S2, and AgGaS2. Other structures included the **Sm2Ti2S2O5 structure** for AgLaOS and the **stannite structure** for (Ag2Sn)0.6000Zn1.2000S3 and Ag1.2Cu0.8ZnSn0.7Ge0.3S4.

Reaction Conditions:

Photocatalytic reactions were conducted in a gas-closed circulation system with visible light irradiation (λ> 420 nm) through a cutoff filter.

Hydrogen Evolution Activity:

The hydrogen evolution rates varied significantly depending on the material and cocatalyst used.

* **AgLaOS:** 8 μmol h-1
* **Ag0.22In0.22Zn1.56S2 (Pt(3%))**: 900 μmol h-1
* **AgGaS2 (Pt(1%))**: 93.6 μmol h-1
* **(AgGa)0.3Zn1.4S2**: 2 μmol h-1
* **AgGaS2**: 637 μmol h-1

* **AgInS2**: 125 µmol h-1
* **(AgIn)0.22Zn1.56S2**: 748 µmol h-1
* **AgGaS2 (Pt(7%))**: 203 µmol h-1
* **(Ag2Sn)0.6000Zn1.2000S3 (Ru(0.5%

From paper findings:
The text describes the synthesis and characterization of AgInS2-ZnS photocatalysts for hydrogen evolution.

Composition: The photocatalysts are composed of AgInS2 and ZnS.

Crystal Structure: The text doesn't explicitly mention the crystal structure type.

Synthesis Method: The text doesn't provide details about the specific synthesis method used.

Reaction Conditions: The photocatalytic hydrogen evolution reaction is carried out under visible-light irradiation using aqueous solutions containing sulfide and sulfite ions as sacrificial electron donors.

Hydrogen Evolution Activity: The AgInS2-ZnS photocatalysts exhibit high activity for hydrogen evolution, even though most sulfide photocatalysts are unstable during the water-splitting reaction. The presence of sacrificial reagents helps to suppress photocorrosion and enhance the stability of the photocatalysts.

【Question】

2. For metal sulfide photocatalysts containing Cu as a constituent element, please specify three important factors strongly affecting the activity for hydrogen evolution.

【Related Information】

From experimental data:
This text describes the synthesis and photocatalytic hydrogen evolution activity of various copper-based sulfide materials.

CuS:

* **Composition:** CuS
* **Crystal Structure:** Covellite
* **Synthesis:** Precursor precipitation followed by heat treatment in a sealed quartz ampule.
* **Reaction Conditions:** Visible light irradiation (λ> 420 nm) in an aqueous solution of K2SO3.
* **Activity:** No H2 evolution observed.

Cu3VS4, Cu3NbS4, Cu3TaS4:

* **Composition:** Cu3VS4, Cu3NbS4, Cu3TaS4
* **Crystal Structure:** Sulvanite
* **Synthesis:** Solid-state reactions of starting materials followed by heat treatment in a sealed quartz ampule.
* **Reaction Conditions:** Visible light irradiation (λ> 420 nm) in an aqueous solution of Na2S and K2SO3.
* **Activity:**
    * Cu3VS4 showed the highest activity, with rates varying depending on the synthesis conditions and the addition of Ru cocatalysts.
    * Cu3NbS4 exhibited moderate activity.
    * Cu3TaS4 showed the lowest activity, even with the addition of a Ru cocatalyst.

Key Findings:

* The Sulvanite structure is favorable for photocatalytic H2 evolution.
* Cu3VS4 is a promising photocatalyst for H2 production.
* Ru cocatalysts significantly enhance the H2 evolution activity of Cu3VS4.

Let me know if you have any other questions.

From paper findings:
This text describes the synthesis and characterization of Cu2SnxGe1−xS3 (CTGS) particles as photocatalysts for hydrogen evolution from water.

Composition: CTGS particles are composed of copper, tin, germanium, and sulfur. The ratio of Sn to Ge can be varied to tune the electronic properties of the material.

Crystal Structure: CTGS particles have a chalcopyrite crystal structure.

Synthesis Method: CTGS particles are synthesized via a solid-state reaction by varying the molar ratio of the precursors.

Reaction Conditions: The photocatalytic hydrogen evolution reaction is performed in an aqueous solution containing Na2S and Na2SO3 as sacrificial reagents under visible light irradiation.

Hydrogen Evolution Activity:

* The incorporation of Ge into the CTGS structure negatively shifts the conduction band minimum, leading to a tunable band gap energy (0.77-1.49 eV).
* This shift increases the driving force for photoexcited electrons involved in hydrogen evolution.
* The addition of a Ru catalyst further enhances the hydrogen evolution rate.
* CTGS particles show good stability even after repeated use.

The text highlights the potential of CTGS particles as a promising photocatalyst for sustainable hydrogen production.

【Question】

3. With respect to AgGaS2, please specify three important factors strongly affecting the activity for hydrogen evolution.

【Related Information】

From experimental data:

AgGaS2 with a Chalcopyrite crystal structure was synthesized via solid-state reactions. The synthesis involved mixing starting materials, sealing them in a quartz ampule, and heat-treating at 1073 K for 10 hours under vacuum.

Photocatalytic hydrogen evolution activity was investigated using a gas-closed circulation system with visible light irradiation ($\lambda > 420$ nm).

The rate of H2 evolution varied depending on the presence and concentration of cocatalysts:

* **Without cocatalyst:** 637 µmol h-1
* **Ir(1%):** 166 µmol h-1
* **Ir(3%):** 251 µmol h-1
* **Ru(1%):** 1150 µmol h-1
* **Ru(2%):** 849 µmol h-1
* **Ru(3%):** 673 µmol h-1

The text suggests that Ru-based cocatalysts, particularly Ru(1%), significantly enhance the hydrogen evolution activity of AgGaS2.

From paper findings:

This text describes the synthesis and photocatalytic activity of nickel-doped AgGaS2 for hydrogen evolution.

Composition: The material is AgGaS2 doped with nickel.

Crystal Structure Type: The text doesn't explicitly state the crystal structure type but mentions that AgGaS2 possesses a higher conduction band than AgInS2.

Synthesis Method: The text doesn't detail the specific synthesis method used for the nickel-doped AgGaS2.

Reaction Conditions: The photocatalytic reaction for hydrogen evolution is carried out in an aqueous solution of Na2S and K2SO3 under simulated sunlight irradiation (AM 1.5).

Hydrogen Evolution Activity:

* Nickel-doped AgGaS2 shows photocatalytic activity for hydrogen evolution even without a co-catalyst.
* The rate of hydrogen evolution is 0.5 L h^{-1} cm^{-2} under simulated sunlight irradiation.
* Doping with nickel improves the photocatalytic performance of AgGaS2 by creating an electron donor level in the forbidden band, enabling visible light absorption and water reduction.
* The apparent quantum yield at 420 nm is 20%.

The text also discusses the influence of other dopants (copper, iron, cobalt) on the photocatalytic activity of AgGaS2.

【Question】

4. For metal sulfide photocatalysts with a chalcopyrite structure, please specify three important factors strongly affecting the activity for hydrogen evolution.

【Related Information】

From experimental data:

This text describes the synthesis and hydrogen evolution activity of various chalcopyrite-structured ternary sulfide photocatalysts.

Synthesis:

All photocatalysts were synthesized via solid-state reactions, involving mixing the starting materials and subsequent heat treatment under vacuum. The specific compositions and heat treatment temperatures varied depending on the desired material.

Crystal Structure:

The crystal structure of all synthesized materials was chalcopyrite, except for Zn2GeS4, which exhibited a defect chalcopyrite structure.

Reaction Conditions:

Photocatalytic reactions were conducted in a gas-closed circulation system using visible light (λ> 420 nm) as the irradiation source. The photocatalysts were dispersed in an aqueous solution of Na2S and K2SO3.

Hydrogen Evolution Activity:

The hydrogen evolution activity of the photocatalysts varied significantly, with rates ranging from 0.7 μmol h-1 to 2358 μmol h-1. The addition of a Ru(0.3%) cocatalyst generally enhanced the hydrogen evolution activity.

Key Findings:

* The composition of the ternary sulfide significantly influenced the hydrogen evolution activity.
* The addition of a Ru cocatalyst significantly improved the photocatalytic performance.
* The chalcopyrite structure is a promising framework for developing efficient visible-light-driven hydrogen evolution photocatalysts.

From paper findings:
This text focuses on the use of metal sulfide semiconductors as photocatalysts for hydrogen evolution from water.

Composition: The text primarily discusses the use of cadmium sulfide (CdS) and other metal sulfide compounds like ZnS, CuInS2, AgInS2, and ZnGa2S4.

Crystal Structure Type: The text mentions both three-dimensional bulk structures (like CdS and ZnS) and layered structures (like K4Nb6O17 and K2La2Ti3O10) as potential candidates for photocatalysts.

Synthesis Method: The text doesn't explicitly detail specific synthesis methods for the metal sulfide photocatalysts.

Reaction Conditions: The photocatalytic hydrogen evolution reaction is typically performed under visible light irradiation in an aqueous solution containing a sacrificial electron donor, such as sodium sulfide (Na2S) and sodium sulfite (Na2SO3). These sacrificial reagents prevent the photocorrosion of the sulfide material and contribute to the hydrogen evolution process.

Hydrogen Evolution Activity: The text highlights the potential of metal sulfide semiconductors for visible light-driven

hydrogen evolution. The use of sacrificial reagents is crucial for suppressing photocorrosion and enhancing the hydrogen evolution activity. The text also mentions the importance of the crystal structure, with layered structures potentially offering advantages due to their ability to separate photogenerated electrons and holes.

【Question】

5. For metal sulfide photocatalysts with a stannite structure, please specify three important factors strongly affecting the activity for hydrogen evolution.

【Related Information】

From experimental data:

This text describes the synthesis and hydrogen evolution activity of various ternary chalcogenide photocatalysts.

General Synthesis Method:

All photocatalysts were synthesized using a solid-state reaction method. Starting materials were mixed with excess amounts of specific elements (Ge, Zn, Sn) and sealed in quartz ampoules under vacuum. The mixtures were then heat-treated at specific temperatures (723 K to 923 K) for durations ranging from 8 to 10 hours.

Crystal Structure:

The majority of the synthesized photocatalysts exhibited the Stannite crystal structure.

Reaction Conditions:

Photocatalytic reactions were conducted in a gas-closed circulation system using a top window Pyrex cell. The photocatalysts were dispersed in an aqueous solution containing Na2S and K2SO3 (or K2SO3 for Sr2CuInO3S). Visible light (λ> 420 nm) was used as the irradiation source.

Hydrogen Evolution Activity:

A Ru(0.5%) cocatalyst was loaded onto the photocatalysts via a photodeposition method to enhance hydrogen evolution. The rate of H2 evolution varied significantly among the different photocatalysts, ranging from 3.8 μmol h-1 for Sr2CuInO3S to 581 μmol h-1 for Ag1.2Cu0.8ZnSn0.4Ge0.6S4.

Note: The text does not provide detailed information about the specific composition of each photocatalyst, only the general formula and the ratios of elements used in the synthesis.

From paper findings:
This article reports on the synthesis and characterization of novel stannite-type complex sulfide photocatalysts (AII-Zn-AIV-S4, where AII = Cu and Ag, AIV = Sn and Ge) for hydrogen evolution under visible-light irradiation.

Composition: The photocatalysts are composed of stannite-type complex sulfides with the general formula AII-Zn-AIV-S4.

Crystal Structure Type: The crystal structure is based on the stannite type.

Synthesis Method: The synthesis method involves a solid-state reaction.

Reaction Conditions: The photocatalytic hydrogen evolution reaction is carried out under visible-light irradiation in an aqueous solution containing sacrificial electron donors (Na2S and Na2SO3).

Hydrogen Evolution Activity: The synthesized photocatalysts exhibit high activity for hydrogen evolution from aqueous solutions containing sacrificial electron donors under visible-light irradiation. The presence of sacrificial reagents prevents photocorrosion of the sulfide photocatalyst. The photocatalysts demonstrate good stability even after repeated use.

【Question】

6. Please enumerate five crystal structures of metal sulfide photocatalysts with a trend of the high activity for hydrogen evolution.

【Related Information】

From experimental data:
This text describes the synthesis, crystal structure, and hydrogen evolution activity of various photocatalysts.

Synthesis Method:

Most photocatalysts were synthesized using solid-state reactions, involving mixing starting materials and heat-treating them in a sealed quartz ampule under vacuum.

Crystal Structure Types:

The crystal structures varied among the materials, including:

* Sm2Ti2S2O5 (Sr2CuInO3S)
* Chalcopyrite (AgIn)0.22Zn1.56S2, (AgGa)0.3Zn1.4S2

* La2S3
* Zinc blende (BP)
* Sr2Mn3Sb2O2#Sr2Mn3As2O2 (Sr2Cu2ZnO2S2)
* Covellite (CuS)
* Sulvanite (Cu3NbS4)
* Chalcopyrite(defect) (Zn2GeS4)

Reaction Conditions:

Photocatalytic reactions were conducted in a gas-closed circulation system with a top window Pyrex cell. The photocatalysts were irradiated with visible light (λ> 420 nm) through a cutoff filter from a 300 W Xe lamp.

Hydrogen Evolution Activity:

The rate of hydrogen evolution varied significantly among the materials, ranging from 0.03 µmol h-1 (Sr2Cu2ZnO2S2) to 748 µmol h-1 ((AgIn)0.22Zn1.56S2).

Key Findings:

* (AgIn)0.22Zn1.56S2 exhibited the highest hydrogen evolution activity.
* CuS did not show activity for hydrogen evolution.
* Ru(0.5%) cocatalyst loading improved the activity of Zn2GeS4.

From paper findings:
This research focuses on developing novel stannite-type complex sulfide photocatalysts (A₂-Zn-AIV-S₄, where AI= Cu and Ag; AIV= Sn and Ge) for efficient hydrogen evolution under visible light irradiation.

Composition: The photocatalysts are composed of stannite-type complex sulfides with copper or silver (AI) and tin or germanium (AIV) as constituent elements.

Crystal Structure Type: The researchers synthesized stannite-type complex sulfide photocatalysts, which possess a specific surface nanostep structure.

Synthesis Method: The synthesis method involves the preparation of the stannite-type complex sulfide photocatalysts.

Reaction Conditions: The photocatalytic hydrogen evolution reaction was performed under visible light irradiation using a mixture of Na₂S and Na₂SO₃ as sacrificial reagents.

Hydrogen Evolution Activity: The synthesized stannite-type complex sulfide photocatalysts exhibited high activity for hydrogen evolution under visible light irradiation. The specific surface nanostep structure and the absence of photocorrosive CdS modifiers contributed to the stability and efficiency of the photocatalysts.

The text emphasizes the importance of the specific surface nanostep structure and the absence of photocorrosive CdS modifiers for achieving high hydrogen evolution activity and stability in these novel stannite-type complex sulfide photocatalysts.

【Question】

7. Please enumerate three cocatalysts of metal sulfide photocatalysts with a trend of the high activity for hydrogen evolution.

【Related Information】

From experimental data:

This text describes the synthesis and photocatalytic hydrogen evolution activity of several sulfide-based materials.

GaS:

* **Composition:** Gallium sulfide (GaS)
* **Crystal Structure:** GaSe structure
* **Synthesis:** Aqueous solution of starting materials purged with N2, precipitated, sealed in a quartz ampule, and heat-treated in vacuo.
* **Reaction Conditions:** Visible light (λ> 420 nm) irradiation in a gas-closed circulation system with a mixed aqueous solution of Na2S and K2SO3. Pt(1%) cocatalyst loaded by photodeposition.
* **Hydrogen Evolution Activity:** 252-633 μmol h-1 depending on the reaction conditions.

Cu3NbS4:

* **Composition:** Copper niobium sulfide (Cu3NbS4)
* **Crystal Structure:** Sulvanite structure
* **Synthesis:** Solid-state reactions of starting materials sealed in a quartz ampule and heat-treated in vacuo at 773-973 K for 8 h.
* **Reaction Conditions:** Visible light (λ> 420 nm) irradiation in a gas-closed circulation system with a mixed aqueous solution of Na2S and K2SO3. Ru(0.3%-0.5%) cocatalyst loaded by photodeposition.
* **Hydrogen Evolution Activity:** 273-564 μmol h-1 depending on the synthesis temperature and Ru cocatalyst loading.

Cu0.54Ag0.36Ga0.54In0.36Zn0.2S2:

* **Composition:** Copper silver gallium indium zinc sulfide (Cu0.54Ag0.36Ga0.54In0.36Zn0.2S2)
* **Crystal Structure:** Chalcopyrite structure
* **Synthesis:** Solid-state reactions of starting materials with excess Ga and Zn, sealed in a quartz ampule, and heat-treated in vacuo at 1023 K for 10 h.
* **Reaction Conditions:** Visible light (λ> 420 nm) irradiation in a gas-closed circulation system with a mixed aqueous solution of Na2S and K2SO3. Ru(0.3%) cocatalyst loaded by photodeposition.
* **Hydrogen Evolution Activity:** 2358 μmol

From paper findings:
This research focuses on developing visible-light-driven sulfide photocatalysts for hydrogen evolution.

Composition: The photocatalysts are based on sulfide materials, with specific surface nanostep structures for enhanced activity.

Crystal Structure Type: The text doesn't explicitly mention the specific crystal structure type of the sulfide photocatalysts.

Synthesis Method: The synthesis method is not detailed in the provided excerpt.

Reaction Conditions: The photocatalytic reactions are driven by visible light (λ > 420 nm).

Hydrogen Evolution Activity: The sulfide photocatalysts exhibit high activity for hydrogen evolution, with cocatalysts (e.g., Pt nanoparticles) playing a crucial role in enhancing the process. The presence of cocatalysts significantly improves the water splitting rates.

The text highlights the importance of cocatalysts in achieving efficient photocatalytic water splitting, particularly for the hydrogen evolution reaction.

【Question】

8. For metal sulfide photocatalysts, please explain the mechanism of which formation of solid-solution affects the activity for hydrogen evolution.

【Related Information】

From experimental data:
This study investigated the hydrogen evolution activity of various photocatalysts.

Photocatalysts and their Structures:

* **Commercial ZrS2:** Did not show activity for H2 evolution.
* **CuS:** Covellite structure, did not show activity for H2 evolution.
* **Commercial BP:** Zinc blende structure, showed a low H2 evolution rate of 0.2 μmol h-1.
* **Fe(4%)-doped ZnS:** Zinc blende structure, did not show activity for H2 evolution.
* **Sr2CuInO3S:** Sm2Ti2S2O5 structure, showed a moderate H2 evolution rate of 3.8 μmol h-1.
* **Mn(0.5%)-doped ZnS, Sn(1%)-doped ZnS, In(1%)-doped ZnS, Sn(4%)-doped ZnS:** All zinc blende structures, did not show activity for H2 evolution.

Synthesis Methods:

Most photocatalysts were synthesized by a similar method:

1. Mixing starting materials in aqueous solution.
2. Purging the solution with N2 gas.
3. Precipitating the precursor.
4. Sealing the precursor in a quartz ampule tube and heat-treating it in vacuo.

Reaction Conditions:

* Gas-closed circulation system with a top window Pyrex cell.
* Photocatalyst powder (0.3 g) dispersed in an aqueous solution of K2SO3.
* Irradiation with visible light (λ> 420 nm) through a cutoff filter from a 300 W Xe lamp.

The results suggest that the crystal structure and dopant type play a significant role in determining the hydrogen evolution activity of the photocatalysts.

From paper findings:
This text describes the synthesis and characterization of a visible-light-driven photocatalyst for hydrogen evolution from an aqueous solution containing sulfide and sulfite.

Composition: The photocatalyst is a solid solution of ZnS–CuInS2–AgInS2.

Crystal Structure Type: The text doesn't explicitly state the crystal structure type but mentions the importance of high crystallinity for efficient photocatalysis.

Synthesis Method: The text doesn't provide details about the specific synthesis method used to prepare the ZnS–CuInS2–AgInS2 solid solution.

Reaction Conditions: The photocatalytic reaction is performed under visible-light irradiation in an aqueous solution containing sulfide (S2-) and sulfite (SO32-) as sacrificial electron donors.

Hydrogen Evolution Activity: The ZnS–CuInS2–AgInS2 solid-solution photocatalyst exhibits high activity for hydrogen evolution under visible-light irradiation. The presence of sulfide and sulfite as sacrificial reagents is crucial for suppressing photocorrosion of the sulfide photocatalyst and promoting hydrogen evolution.

The text emphasizes the importance of high crystallinity and surface nanostep structure for efficient photocatalysis. It also highlights the role of sacrificial reagents in suppressing photocorrosion and promoting hydrogen evolution.

【Question】

9. Please explain the reason why it is difficult to employ a metal sulfide photocatalyst as a single particle for water splitting.

【Related Information】

From experimental data:

This text describes the synthesis and photocatalytic hydrogen evolution activity of several chalcogenide materials.

Key findings:

* **(AgIn)0.22Zn1.56S2:** This material exhibits promising hydrogen evolution activity, with rates up to 754 µmol h-1, depending on the synthesis method and cocatalyst loading (Pt). Different synthesis methods and cocatalysts (Pt, Ru) were explored, resulting in varying hydrogen evolution rates.
* **Cu3TaS4:** This material showed lower hydrogen evolution activity (36.8 µmol h-1) compared to (AgIn)0.22Zn1.56S2, likely due to its different crystal structure and electronic properties.
* **AgGaS2:** This material also demonstrated hydrogen evolution activity (93.6 µmol h-1) with a Pt cocatalyst.

General Synthesis Methods:

* **Solid-state reactions:** This method involves mixing starting materials and heating them under controlled conditions to form the desired compound.

* **Precipitation:** An aqueous solution containing starting materials is treated to precipitate the desired compound.
* **Heat treatment:** Precursors are heated under specific conditions (temperature, atmosphere) to transform them into the final product.

Crystal Structures:

* **Chalcopyrite:** This structure is common in many chalcogenide materials, including (AgIn)0.22Zn1.56S2 and AgGaS2.
* **Wurtzite:** This structure is also common in chalcogenides and was observed in some (AgIn)0.22Zn1.56S2 samples.
* **Sulvanite:** This structure is characteristic of Cu3TaS4.

Reaction Conditions:

* **Photocatalytic reactions:** These reactions are typically conducted in a gas-closed circulation system with visible light irradiation.
* **Cocatalysts:** Various cocatalysts (Pt, Ru) are used to enhance the photocatalytic activity.

Hydrogen Evolution Activity:

* The hydrogen evolution rates vary significantly depending on the material, synthesis method, cocatalyst, and reaction conditions.

This summary highlights the key aspects of the text, providing a concise overview of the materials, their synthesis, and their photocatalytic performance.

From paper findings:
This text focuses on the challenges and advancements in photocatalytic water splitting using metal sulfide photocatalysts.

Composition: The text primarily discusses metal sulfide photocatalysts, particularly focusing on ZnS–CuInS2–AgInS2 solid-solution and other metal sulfides like CdS and CuI-containing metal sulfides.

Crystal Structure Type: The text mentions layered perovskite structure for oxysulfide photocatalysts like Sm 2Ti2O5S2.

Synthesis Method: While not explicitly detailed, the text implies the synthesis of solid-solution photocatalysts and the use of various metal sulfides as photocatalysts.

Reaction Conditions: The text highlights the need for visible light irradiation and the use of sacrificial reagents in some cases.

Hydrogen Evolution Activity: The text emphasizes the challenges of photocorrosion in metal sulfide photocatalysts and proposes two strategies to overcome this:

1. **Z-scheme system:** Combining a H2-evolving metal sulfide photocatalyst with an O2-evolving photocatalyst and an electron mediator.
2. **Photoelectrochemical cell:** Utilizing metal sulfides as p-type photocathodes for water reduction to hydrogen.

The text concludes by emphasizing the ongoing research and development efforts in this field, aiming to develop efficient and stable photocatalysts for sustainable hydrogen production.

【Question】
10. Please specify several examples of elements constituting the valence band of metal sulfide photocatalysts.

【Related Information】
From experimental data:
This text describes the synthesis and hydrogen evolution activity of various copper-based sulfide photocatalysts.

General Synthesis Method:

Most photocatalysts were synthesized via solid-state reactions, involving mixing starting materials and heating them in a sealed quartz tube under vacuum.

Crystal Structures:

* **Sulvanite structure:** Cu3NbS4
* **Chalcopyrite structure:** (AgIn)0.22Zn1.56S2, (AgGa)0.3Zn1.4S2, (CuIn)0.09Zn1.82S2

Reaction Conditions:

Photocatalytic reactions were conducted in a gas-closed circulation system with visible light irradiation (λ> 420 nm) using a 300 W Xe lamp. The photocatalysts were dispersed in an aqueous solution of Na2S and K2SO3.

Hydrogen Evolution Activity:

The hydrogen evolution rates varied significantly depending on the specific composition and synthesis conditions.

* **(AgIn)0.22Zn1.56S2** exhibited the highest activity (748 µmol h-1).
* **Cu3NbS4** showed moderate activity, with the addition of a Ru(0.5%) cocatalyst significantly enhancing its performance.
* **CuS** did not show any activity for hydrogen evolution.

Key Findings:

* The crystal structure and composition significantly influence the hydrogen evolution activity of copper-based sulfide photocatalysts.
* The addition of a cocatalyst, such as Ru, can significantly improve the photocatalytic performance.
* Further optimization of synthesis conditions and catalyst design is needed to enhance the efficiency of these materials for hydrogen production.

From paper findings:
This text discusses metal sulfide photocatalysts for visible light-driven hydrogen evolution.

Composition:

The text focuses on metal sulfides, particularly those containing Cu, Bi, Pb, and Sn, which contribute to forming valence bands with more negative levels than O 2p orbitals, leading to narrower band gaps.

Crystal Structure Type:

The text mentions the Aurivillius structure for a specific example.

Synthesis Method:

The text doesn't explicitly describe a specific synthesis method for the metal sulfide photocatalysts.

Reaction Conditions:

The text mentions using a solar simulator with an air-mass 1.5 filter (AM-1.5) irradiating at 100 mW cm⁻². It also highlights the importance of a sacrificial reagent (e.g., Ag⁺) to suppress photocorrosion.

Hydrogen Evolution Activity:

The text emphasizes the potential of metal sulfide photocatalysts for visible light-driven H₂ evolution. It cites examples like Pt/CdS and CuAgZnSnS₄, demonstrating their activity. The text also discusses the role of Cu, Bi, Pb, and Sn ions in forming valence bands and contributing to the photocatalytic activity.

【Question】

11. Please specify several examples of elements constituting the conduction band of metal sulfide photocatalysts.

【Related Information】

From experimental data:

This text describes the synthesis, crystal structure, and hydrogen evolution activity of various sulfide-based photocatalysts.

Synthesis Method:

All photocatalysts were synthesized using solid-state reactions. Starting materials were mixed, sealed in quartz ampoules, and heated under vacuum for a specific duration and temperature.

Crystal Structure Type:

The crystal structures varied, with Chalcopyrite being the most common, followed by Sulvanite and Sm2Ti2S2O5.

Reaction Conditions:

Photocatalytic reactions were conducted in a gas-closed circulation system with a top window Pyrex cell. The photocatalysts were dispersed in aqueous solutions containing Na2S, K2SO3, or K2SO3, and irradiated with visible light (λ> 420 nm) through a cutoff filter from a 300 W Xe lamp.

Hydrogen Evolution Activity:

The hydrogen evolution rates varied significantly depending on the photocatalyst composition. Some materials, like (AgIn)0.22Zn1.56S2 and Cu0.54Ag0.36Ga0.36In0.54Zn0.2S2, exhibited high activity (748 and 1168 µmol h-1, respectively), while others, like CuS and Cu2GeGa6S12, showed low activity or no activity.

Cocatalysts:

In some cases, cocatalysts like Ru(0.5%) were loaded onto the photocatalysts via photodeposition to enhance hydrogen evolution activity.

From paper findings:

This text focuses on metal sulfide photocatalysts for visible-light-driven hydrogen evolution.

Composition:

* Primarily composed of metal cations with d0 and d10 electronic configurations (e.g., Cu, Ag, Zn, Sn) and sulfur.
* Often incorporate Cu(I) ions for p-type semiconductor character and visible light absorption.
* Solid solutions and doping with other elements are used to tune band gap and photocatalytic activity.

Crystal Structure Type:

* Primarily chalcopyrite-type structures (e.g., Cu2ZnSnS4, CuAgZnSnS4).

Synthesis Method:

* Not explicitly detailed in the provided text.

Reaction Conditions:

* Aqueous solution containing a sacrificial electron donor (e.g., Na2S/Na2SO3) to suppress photocorrosion.
* Irradiation with a solar simulator (AM-1.5) with a power of 100 mW cm-2.

Hydrogen Evolution Activity:

* Metal sulfide photocatalysts with narrow band gaps (e.g., CuAgZnSnS4) exhibit photocatalytic activity for H2 evolution.
* Cu(I) incorporation and solid solutions enhance visible light absorption and photocatalytic performance.
* Photocorrosion is suppressed by using sacrificial electron donors.

Let me know if you have any other questions.

【Question】

12. Please specify metal sulfide photocatalysts with response to long wavelength of visible light up to 600 nm and explain the reason why those photocatalysts can response to long wavelength of visible light in terms of their band structures.

【Related Information】

Related Information:

From experimental data:
This text describes the synthesis, crystal structure, and hydrogen evolution activity of several chalcogenide-based photocatalysts.

Synthesis:

All photocatalysts were synthesized primarily using solid-state reactions, involving mixing starting materials and subsequent heat treatment under vacuum. Some variations included using excess amounts of certain elements, controlled atmosphere (N2 flow), and precipitation methods to prepare precursors.

Crystal Structure:

The crystal structures varied depending on the composition. Common structures included Chalcopyrite, Wurtzite, and Sulvanite.

Reaction Conditions:

Photocatalytic reactions were conducted in a gas-closed circulation system with a Pyrex cell. The photocatalysts were irradiated with visible light (λ> 420 nm) through a cutoff filter from a 300 W Xe lamp.

Hydrogen Evolution Activity:

The rate of hydrogen evolution varied significantly between the different photocatalysts, ranging from 0.03 µmol h-1 to 748 µmol h-1.

Key Findings:

* (AgIn)0.22Zn1.56S2 and Cu3NbS4 exhibited the highest hydrogen evolution activity.
* The crystal structure and composition significantly influenced the photocatalytic performance.
* The use of a Pt cocatalyst on (AgIn)0.22Zn1.56S2 further enhanced its activity.

From paper findings:
This text discusses metal sulfide photocatalysts for visible light-driven hydrogen evolution.

Composition:

The text focuses on various metal sulfide compositions, including CdS, ZnS, (CuIn)xZn2(1-x)S2, (CuAg)xIn2xZn2(1-2x)S2, (Cu xAg1-x)2ZnSnS4, and La5Ti2Cu(S1-xSex)5O7.

Crystal Structure Type:

The text mentions both bulk structures (like CdS and ZnS) and layered structures (like K4Nb6O17 and K2La2Ti3O10) as potential candidates for photocatalysts.

Synthesis Method:

The text doesn't explicitly detail specific synthesis methods for these materials.

Reaction Conditions:

The text highlights the importance of using sacrificial reagents (like Na2S/Na2SO3) to suppress photocorrosion and enhance hydrogen evolution activity. It also mentions the need for specific reaction conditions to prevent the degradation of metal sulfide photocatalysts at low pH.

Hydrogen Evolution Activity:

The text emphasizes the potential of metal sulfide photocatalysts for visible light-driven hydrogen evolution. It discusses the role of band engineering, crystal structure, and particle size in influencing the photocatalytic activity.

【Question】

13. For metal sulfide photocatalysts, please specify common features of elements constituting valence band and conduction band, respectively.

【Related Information】

From experimental data:

This text describes the synthesis and hydrogen evolution activity of several chalcopyrite and sulvanite-type photocatalysts.

Synthesis Method:

All photocatalysts were synthesized via solid-state reactions. Starting materials were mixed, sealed in quartz ampoules, and heated under vacuum for a specific duration and temperature.

Crystal Structure:

* **Chalcopyrite Structure:** (AgGa)0.3Zn1.4S2, (AgIn)0.22Zn1.56S2, Cu0.54Ag0.36Ga0.36In0.54Zn0.2S2, Cu0.54Ag0.36Ga0.54In0.36Zn0.2S2, Cu0.54Ag0.36Ga0.81In0.09Zn0.2S2, Cu0.81Ag0.09Ga0.54In0.36Zn0.2S2
* **Sulvanite Structure:** Cu3NbS4

Reaction Conditions:

Photocatalytic reactions were conducted in a gas-closed circulation system with a top window Pyrex cell. The photocatalysts were dispersed in a mixed aqueous solution of Na2S and K2SO3 and irradiated with visible light (λ> 420 nm) through a cutoff filter from a 300 W Xe lamp.

Hydrogen Evolution Activity:

The rate of H2 evolution varied significantly depending on the specific composition and the presence of a Ru cocatalyst.

* **Highest Activity:** Cu0.54Ag0.36Ga0.54In0.36Zn0.2S2 (Ru(0.5%)-loaded) with 2358 μmol h-1
* **Lowest Activity:** (AgGa)0.3Zn1.4S2 with 2 μmol h-1

The text highlights the importance of composition and cocatalyst loading in optimizing the hydrogen evolution activity of these photocatalysts.

From paper findings:
This text focuses on metal sulfide photocatalysts for visible-light-driven hydrogen evolution.

Composition:

The text highlights the use of metal ions like Cu, Ag, Bi, Pb, and Sn, often in combination with sulfur, to form the photocatalyst material. The presence of these metals, particularly those with d10 electron configurations, contributes to the formation of the conduction band.

Crystal Structure Type:

The text mentions the use of metal sulfides with a Z-scheme system and as photocathodes in photoelectrochemical cells. It also discusses the use of CuI-containing metal sulfides due to their narrow band gaps.

Synthesis Method:

The text doesn't explicitly describe a specific synthesis method for the metal sulfide photocatalysts.

Reaction Conditions:

The text mentions the use of a solar simulator with an air-mass 1.5 filter (AM-1.5) irradiating at 100 mW cm⁻² as a light source for the photocatalytic reactions.

Hydrogen Evolution Activity:

The text emphasizes the potential of metal sulfide photocatalysts for visible-light-driven hydrogen evolution. It cites examples like CuAgZnSnS₄ with a narrow band gap (1.4 eV) showing photocatalytic activity for H₂ evolution. The text also discusses the role of p-type metal sulfides as photocathodes in photoelectrochemical systems.

【Question】

14. Please specify the reason why development of metal sulfide photocatalysts with respond to long wavelength of visible light is important for practical solar water splitting.

【Related Information】

From experimental data:

This text describes the synthesis, crystal structure, and hydrogen evolution activity of various photocatalysts.

General Synthesis Method:

Most photocatalysts were synthesized using solid-state reactions, involving mixing starting materials and heat-treating them in a sealed quartz ampule under vacuum.

Crystal Structures:

The crystal structures varied depending on the composition, with common types including Chalcopyrite, Wurtzite, Sulvanite, and Sm2Ti2S2O5.

Reaction Conditions:

Photocatalytic reactions were conducted in a gas-closed circulation system with a Pyrex cell. The photocatalysts were irradiated with visible light (λ> 420 nm) through a cutoff filter from a 300 W Xe lamp.

Hydrogen Evolution Activity:

The hydrogen evolution activity varied significantly among the photocatalysts. (AgIn)0.22Zn1.56S2 with a Pt cocatalyst showed the highest activity, while CuS and Sr2Cu2ZnO2S2 exhibited very low activity.

Key Findings:

* The composition and crystal structure significantly influence the hydrogen evolution activity.
* Pt cocatalysts can significantly enhance the activity of certain photocatalysts.
* Some materials, like CuS, show no activity for hydrogen evolution.

Let me know if you have any other questions.

From paper findings:
This text discusses the development of visible-light-driven photocatalysts for water splitting, focusing on metal sulfide materials.

Composition:

The text highlights the use of metal sulfide solid solutions, such as ZnS–CuInS2–AgInS2, as promising photocatalysts.

Crystal Structure Type:

The text mentions the sulvanite structure of Cu3MS4 (M = V, Nb, Ta) as another potential photocatalyst.

Synthesis Method:

The text doesn't explicitly detail specific synthesis methods for the mentioned photocatalysts.

Reaction Conditions:

The text emphasizes the need for visible light irradiation and, in some cases, sacrificial reagents (e.g., S2− and SO32−) to facilitate the photocatalytic reaction.

Hydrogen Evolution Activity:

The text reports the successful demonstration of hydrogen evolution from aqueous solutions containing sulfide and sulfite over a ZnS–CuInS2–AgInS2 solid-solution photocatalyst. It also mentions the activity of Cu3MS4 for sacrificial hydrogen evolution under visible light.

The text primarily focuses on the challenges and opportunities associated with developing efficient visible-light-driven photocatalysts for water splitting, particularly emphasizing the role of metal sulfide materials.

Box S4. Public URLs of output responses generated using GPT-4o for the 14 expert-defined questions.

【Question】

1. For metal sulfide photocatalysts containing Ag as a constituent element, please specify three important factors strongly affecting the activity for hydrogen evolution.

【Public URL of output responses generated using GPT-4o】

https://chatgpt.com/s/t_68dca111d99c8191a97ba600c33847c0

【Question】

2. For metal sulfide photocatalysts containing Cu as a constituent element, please specify three important factors strongly affecting the activity for hydrogen evolution.

【Public URL of output responses generated using GPT-4o】

https://chatgpt.com/s/t_68dca15f4c608191bd5cbe4c1121fb00

【Question】

3. With respect to AgGaS2, please specify three important factors strongly affecting the activity for hydrogen evolution.

【Public URL of output responses generated using GPT-4o】

https://chatgpt.com/s/t_68dca17851308191a154772f5f330bae

【Question】

4. For metal sulfide photocatalysts with a chalcopyrite structure, please specify three important factors strongly affecting the activity for hydrogen evolution.

【Public URL of output responses generated using GPT-4o】

https://chatgpt.com/s/t_68dca1937a8881919af42bffad524684

【Question】

5. For metal sulfide photocatalysts with a stannite structure, please specify three important factors strongly affecting the activity for hydrogen evolution.

【Public URL of output responses generated using GPT-4o】

https://chatgpt.com/s/t_68dca1a9c9608191b2e12162e8efc638

【Question】

6. Please enumerate five crystal structures of metal sulfide photocatalysts with a trend of the high activity for hydrogen evolution.

【Public URL of output responses generated using GPT-4o】
https://chatgpt.com/s/t_68dca1c89cb08191ad99accb5b414aa2

【Question】
7. Please enumerate three cocatalysts of metal sulfide photocatalysts with a trend of the high activity for hydrogen evolution.

【Public URL of output responses generated using GPT-4o】
https://chatgpt.com/s/t_68dca3c665c48191bd8ac30418b2d7cb

【Question】
8. For metal sulfide photocatalysts, please explain the mechanism of which formation of solid-solution affects the activity for hydrogen evolution.

【Public URL of output responses generated using GPT-4o】
https://chatgpt.com/s/t_68dca1e0eb8881919b1bcc7444a391c2

【Question】
9. Please explain the reason why it is difficult to employ a metal sulfide photocatalyst as a single particle for water splitting.

【Public URL of output responses generated using GPT-4o】
https://chatgpt.com/s/t_68dca37637d08191a980fecee4edc1f8

【Question】
10. Please specify several examples of elements constituting the valence band of metal sulfide photocatalysts.

【Public URL of output responses generated using GPT-4o】
https://chatgpt.com/s/t_68dca1f780348191a260e7c8a22ec991

【Question】
11. Please specify several examples of elements constituting the conduction band of metal sulfide photocatalysts.

【Public URL of output responses generated using GPT-4o】
https://chatgpt.com/s/t_68dca210f3548191ade37ffb4dad9d78

【Question】

12. Please specify metal sulfide photocatalysts with response to long wavelength of visible light up to 600 nm and explain the reason why those photocatalysts can response to long wavelength of visible light in terms of their band structures.

【Public URL of output responses generated using GPT-4o】

https://chatgpt.com/s/t_68dca25bf8248191aca70ce925d4697c

【Question】

13. For metal sulfide photocatalysts, please specify common features of elements constituting valence band and conduction band, respectively.

【Public URL of output responses generated using GPT-4o】

https://chatgpt.com/s/t_68dca2b71a7c8191a3f64ffb9e5894f4

【Question】

14. Please specify the reason why development of metal sulfide photocatalysts with respond to long wavelength of visible light is important for practical solar water splitting.

【Public URL of output responses generated using GPT-4o】

https://chatgpt.com/s/t_68dca3073c2c8191a80e236ebda32798
